# Weaned beef calves fed selenium-biofortified alfalfa hay have an enriched nasal microbiota compared with healthy controls

**DOI:** 10.1371/journal.pone.0179215

**Published:** 2017-06-08

**Authors:** Jean A. Hall, Anitha Isaiah, Charles T. Estill, Gene J. Pirelli, Jan S. Suchodolski

**Affiliations:** 1 Department of Biomedical Sciences, College of Veterinary Medicine, Oregon State University, Corvallis, Oregon, United States of America; 2 Gastrointestinal Laboratory, College of Veterinary Medicine, Department of Small Animal Clinical Sciences, Texas A&M University, College Station, Texas, United States of America; 3 Department of Clinical Sciences, College of Veterinary Medicine, Oregon State University, Corvallis, Oregon, United States of America; 4 Department of Animal and Rangeland Sciences, College of Agricultural Sciences, Oregon State University, Corvallis, Oregon, United States of America; Ross University School of Veterinary Medicine, SAINT KITTS AND NEVIS

## Abstract

Selenium (Se) is an essential trace mineral important for immune function and overall health of cattle. The nasopharyngeal microbiota in cattle plays an important role in overall respiratory health, especially when stresses associated with weaning, transport, and adaptation to a feedlot affect the normal respiratory defenses. Recent evidence suggests that cattle diagnosed with bovine respiratory disease complex have significantly less bacterial diversity. The objective of this study was to determine whether feeding weaned beef calves Se-enriched alfalfa (*Medicago sativa*) hay for 9 weeks in a preconditioning program prior to entering the feedlot alters nasal microbiota. Recently weaned beef calves (n = 45) were blocked by sex and body weight, randomly assigned to 3 treatment groups with 3 pens of 5 calves per treatment group, and fed an alfalfa hay based diet for 9 weeks. Alfalfa hay was harvested from fields fertilized with sodium selenate at a rate of 0, 45.0 or 89.9 g Se/ha. Blood samples were collected biweekly and analyzed for whole-blood Se concentrations. Nasal swabs were collected during week 9 from one or two calves from each pen (total n = 16). Calculated Se intake from dietary sources was 3.0, 15.6, and 32.2 mg Se/head/day for calves consuming alfalfa hay with Se concentrations of 0.34 to 2.42 and 5.17 mg Se/kg dry matter, respectively. Whole-blood Se concentrations after 8 weeks of feeding Se-fertilized alfalfa hay were dependent upon Se-application rates (0, 45.0, or 89.9 g Se/ha) and were 155, 345, and 504 ng/mL (*P*_Linear_ < 0.0001). Microbial DNA was extracted from nasal swabs and amplified and sequenced. Alpha rarefaction curves comparing the species richness (observed OTUs) and overall diversity (Chao1, Observed OTU, and Shannon index) between calves fed selenium-biofortified alfalfa hay compared with control calves showed that Se-supplementation tended to be associated with an enriched nasal microbiota. ANOSIM of unweighted UniFrac distances showed that calves fed high Se-biofortified alfalfa hay clustered separately when compared with control calves in the PCoA plot (*R* = 0.216, *P* = 0.04). The bacterial orders *Lactobacillales* and *Flavobacteriales* were increased in healthy control calves compared with *Clostridiales* and *Bacteroidales* being increased in calves fed Se-biofortified alfalfa hay. Although there were strong trends, no significant differences were noted for any of the bacterial taxa. Based upon these findings, we suggest that weaned beef calves fed Se-biofortified hay tend to have an enriched nasal microbiota. Feeding Se-biofortified alfalfa hay to weaned beef calves prior to entering the feedlot is a strategy for increasing nasopharyngeal microbial diversity.

## Introduction

Selenium (Se) is an essential trace mineral important for immune function and overall health of cattle. Se deficiency can cause nutritional myodegeneration, also known as white muscle disease, characterized by muscle weakness, heart failure, unthriftiness, and death. Insufficient Se intake can cause subclinical diseases resulting in poor livestock performance. The role of Se in animal health is based primarily on the functions of selenocysteine-containing proteins, many of which have antioxidant activities [1]. Oxidative stress is a primary mechanism by which reactive oxygen species (ROS) influence biological processes and may contribute to the pathogenesis of disease by modifying the expression of proinflammatory genes. The beneficial effects of Se supplementation may result from selenoproteins converting harmful ROS to less reactive molecules, as well as influencing the expression of redox-regulated genes [2].

In Se-deficient areas, several means of Se supplementation are available, e.g., organic Se-yeast can be added to feed, or inorganic Na selenite can be added to mineral mixes for free-choice consumption. Agronomic biofortification is an alternative approach, whereby the Se concentration of forage is increased through the use of Se-containing fertilizer amendments [3]. We have demonstrated that Se-fertilization is an effective strategy for Se supplementation in sheep [4, 5]. Our goal was to apply this Se-supplementation strategy to weaned beef calves to optimize cattle health.

Conversely, Se toxicity and livestock death can occur when cattle consume excessive amounts of Se in their diet. For example, consumption of Se-accumulator plants, including species of *Astragalus*, *Brassica*, *and Stanleya*, which survive in soils with high Se concentrations, can cause toxicity. Although cattle do not typically eat these plants, care should be taken when grazing in areas where they grow. Death can also occur when excessive amounts of inorganic Se are injected. The majority of published experimental work on selenosis in ruminants (reviewed in [6]) is based on studies using inorganic forms of Se, such as sodium selenate or sodium selenite, whereas the predominant chemical form of Se in forage is selenomethionine [7]. In some cattle, chronic dietary exposure for 4 months to 0.28 and 0.8 mg Se/kg in the form of selenomethionine resulted in mild (subclinical) to severe (clinical) forms of alkali disease [6]. Selenium is regulated as a feed additive by the Food and Drug Administration because of its potential toxic effects. In Oregon, the use of Se as a soil amendment is not regulated. Thus, Se deficiency in cattle, caused by low Se intake, can be mitigated by increasing the Se concentrations in hay fed to cattle.

Dietary glutamine supplementation has been shown to regulate intestinal immunity, for example though enhancement of secretory IgA production, and studies in mice show that glutamine supplementation acts in part by altering the intestinal bacterial community [8, 9]. It is thought that functional amino acids regulate immunity by modulating gene expression, protein synthesis, and cellular signaling (reviewed in [8]). We have previously shown that Se supplementation alters gene expression profiles associated with innate immunity in circulating neutrophils of sheep. Thus, we hypothesized that Se supplementation in cattle might also alter bacterial communities.

Similar to the role of the intestinal microbiome in gastrointestinal health, the respiratory microbiome is a strong determinant of respiratory health [10]. The nasal microbial ecosystem primes the immune system and provides colonization resistance against acquisition of new pathogens, plus it contains potential pathogenic bacteria living among harmless commensals [10]. There is recent evidence to suggest that the nasopharyngeal microbiota plays an important role in respiratory health in cattle [11, 12]. Cattle with bovine respiratory disease (BRD) complex had significantly less bacterial diversity and fewer Operational Taxonomic Units (OTUs) in their nasopharynx [11]. Reduced diversity of nasopharyngeal microbiota may be a risk factor to BRD development [11]. The nasopharyngeal microbiota of beef cattle undergoes major evolution from weaning to 40 days after arrival at a feedlot [12]. Hypothetically, this may explain why cattle are most susceptible to bovine respiratory disease (BRD) complex during transition to a feedlot environment. The question thus arises, beyond the use of antibiotics and vaccines, might disease prevention be achieved by altering environmental factors to impact the success of specific bacterial species in this territorial competition [13].

The objective of this study was to determine whether feeding weaned beef calves Se-enriched alfalfa (*Medicago sativa*) hay for 8 weeks in a preconditioning program prior to entering the feedlot alters the nasal microbiota. We hypothesized that feeding weaned beef calves forage fertilized with increasing amount of Na-selenate would improve the overall diversity of the nasal microbiome.

## Methods

### Animal ethics statement and study design

The experimental protocol was reviewed and this study was approved by the Oregon State University Animal Care and Use Committee (ACUP Number: 4705). This was a prospective clinical trial of 9-week duration (October 10, 2015 through December 10, 2015) involving 45 weaned beef calves, primarily of Angus breeding. The study design consisted of 3 treatment groups, with three pens of five animals per treatment. The study was conducted at the Steer Metabolism barn on the Oregon State University campus (Corvallis, OR, USA).

Corvallis is located at an elevation of 72 m within the Marine West Coast climate zone. Temperatures are mild year round, with warm, sunny summers and mild, wet winters with persistent overcast skies. Because of its close proximity to the coast range, temperatures dropping below freezing are uncommon. Average monthly temperatures for November are 10.8°C (high) and 3.3°C (low). Rainfall total is 110.9 cm/yr. Typical distribution of precipitation includes about 50% of the annual total from December through February, lesser amounts in the spring and fall, and very little during summer.

The weaned beef calves ranged in age from 6.5 to 9 months (222 ± 6.4 days; mean ± SEM) and originated from the Oregon State University beef ranch, Corvallis, OR, USA. Body weights at weaning ranged from 240 to 334 kg (286 ± 9.3 kg, mean ± SEM), and body condition scores ranged from 6 to 7 (1 to 9 scale). There were 17 heifers and 28 steer calves in the study. Routine farm management practices, including vaccinations and deworming, were the same for all treatment groups.

Using a randomized complete block design, calves were blocked at the time of weaning by sex and body weight and then assigned to one of 3 treatment groups of 15 calves each. Ear tags were used to identify calves. Calves were then placed by treatment group into pens (3 pens of 5 calves/treatment group). The pens were 14 m^2^/calf; concrete flooring in open lots that were strip cleaned once weekly; shavings in loafing area with 4.5 m^2^/calf; concrete bunks with 98 cm of feeder space/calf; all measurements exceeded requirements [14] with continuous access to water, feed bunks, and shelter.

Calves were fed 70% alfalfa hay and 30% grass hay twice daily. The amount of hay fed was adjusted weekly to ensure that calves had all they wanted for consumption yet with minimal wastage. The ration was formulated for growing beef calves in the 250 to 350 kg weight range to achieve a target average daily gain of 0.5 kg/day. The goal was to feed hay at a rate of 70% alfalfa and 30% grass hay. Calves were transitioned to their respective alfalfa hay sources over a 7 day period. Alfalfa hay was fed as follows: 0.68 kg/head/day 1; 1.14 kg/head/day 2; 1.59 kg/head day 3; 2.27 kg/head/day 4; 2.95 kg/head/day 5; 3.41 kg/head/day 6; and 3.86 kg/head/day 7. During this first week, grass hay was added to achieve a total hay intake of 5.45 kg/head/days 1 and 2; 5.91 kg/head/days 3 and 4; 6.36 kg/head/days 5 and 6; and 6.82 kg/head/days 7 and 8. Thereafter, calves were fed 70% alfalfa hay and 30% grass hay with increases as needed to provide all the hay they wanted for consumption yet with minimal wastage. By week 9, calves were consuming between 8.18 to 9.09 kg hay/head/day.

Prior to this study, calves had free-choice access to a mineral supplement containing 120 mg/kg Se from sodium-selenite. The mineral supplement (dry matter basis) was in loose granular format and contained 57.0 to 64.0 g/kg calcium; 30.0 g/kg phosphorus; 503 to 553 g/kg salt (NaCl); 50.0 g/kg magnesium; 50 mg/kg cobalt; 2,500 mg/kg copper; 200 mg/kg manganese; 200 mg/kg iodine; 6,500 mg/kg zinc (Wilbur-Ellis Company, Clackamas, OR). During this feeding trial, mineral supplement was added as a top dressing to the hay for each pen of calves at the rate of 0.1 mg Se/kg of dry matter intake [15]. At the beginning of the study this calculation was 0.49 mg Se/head/day, or 4.08 g mineral supplement/head/day.

### Selenium fortified-alfalfa hay

The soil was enriched with Se by mixing inorganic sodium-selenate (RETORTE Ulrich Scharrer GmbH, Röthenbach, Germany) with water and spraying it onto the soil surface of an alfalfa field at application rates of 0, 45.0, or 89.9 g Se/ha immediately after the second cutting of hay in July 2014. The application rates were chosen based on a previous study [3]. Third-cutting alfalfa hay was harvested 40 days after Se application and then analyzed for nutrient and Se content. Alfalfa yield was approximately 4.9 ton/ha. A Penn State forage sampler was used to take 25 cores from random bales in each hay source (0, 45.0, or 89.9 g Se/ha). Samples were collected prior to beginning the feeding trial for each alfalfa hay source. Core samples were mixed well and representative samples selected for analysis. Alfalfa hay samples were submitted to commercial laboratories for routine nutrient analysis (Table 1; Cumberland Analytical Services, Maugansville, MD) and Se analysis (Utah Veterinary Diagnostic Laboratory, Logan, UT). Alfalfa hay dry matter determination was completed at a temperature of 105°C for 12 to 14 h in a forced draught oven. Methods for crude protein (CP), acid detergent fiber (ADF), ash, and minerals were performed according to the Association of Official Analytical Chemists [16]. The neutral detergent fiber (NDF) was determined according to Van Soest et al. [17]. Soluble protein was determined according to Krishnamoorthy et al. [18]. Plant samples were prepared for Se analysis as previously described [19], and Se determined using inductively coupled argon plasma emission spectroscopy (ICP-MS; ELAN 6000, Perkin Elmer, Shelton, CT). Quantification of Se was performed by the standard addition method, using a 4-point standard curve. A quality-control sample (in similar matrix) was analyzed after every 5 samples, and analysis was considered acceptable if the Se concentration of the quality-control sample fell within ± 5% of the standard/reference value for the quality control.

**Table 1:** Alfalfa and grass hay nutrient compositions (dry matter basis)^1^^–^^3^.

Nutrient	Alfalfa Hay			Grass Hay
	Control	Med-Se	High-Se	
Dry matter, g/kg	874	874	878	954
Crude protein, g/kg	170	183	157	78
Acid detergent fiber, g/kg	377	386	373	380
Neutral detergent fiber, g/kg	402	417	430	604
Nonfiber carbohydrates, g/kg	316	289	286	243
Fat, g/kg	13.7	12.4	12.0	0.7
Ash, g/kg	98.3	98.6	115.0	74.3
TDN, g/kg	600	596	564	588
Calcium, g/kg	13.3	13.1	13.4	4.1
Phosphorus, g/kg	2.4	2.6	2.5	2.1
Magnesium, g/kg	3.2	3.5	3.4	1.8
Potassium, g/kg	23.3	23.3	22.5	14.5
Sodium, g/kg	0.98	1.14	1.11	1.91
Copper, mg/kg	10	11	11	5
Iron, mg/kg	292	447	261	73
Manganese, mg/kg	39	48	41	311
Zinc, mg/kg	20	19	20	22
Selenium, mg/kg	0.34	2.42	5.17	0.36

^1^Alfalfa hay samples were submitted to Cumberland Analytical Services, Maugansville, MD for routine nutrient analysis and to Utah Veterinary Diagnostic Laboratory, Logan, UT for Se analysis.

^2^Alfalfa hay DM determination was completed at a temperature of 105°C for 12 to 14 h in a forced draught oven. Methods for CP, ADF, ash, and minerals were performed according to the Association of Official Analytical Chemists [16]. The NDF was determined according to Van Soest et al. [17]. Soluble protein was determined according to Krishnamoorthy et al. [18].

^3^Alfalfa hay samples were prepared for Se analysis as described by Davis et al. [19], and Se determined using inductively coupled argon plasma emission spectroscopy (ICP-MS; ELAN 6000, Perkin Elmer, Shelton, CT).

### Blood collection for selenium analyses

Blood samples were collected from the jugular vein of weaned beef calves, at baseline and after 2, 4, 6 and 8 weeks of alfalfa hay consumption, into evacuated EDTA tubes (2 mL; final EDTA concentration 2 g/L; Becton Dickinson, Franklin Lakes, NJ) and stored on ice until they were frozen at -20°C to measure whole blood Se (WB-Se) concentrations. Selenium concentrations were determined by a commercial laboratory (Utah Veterinary Diagnostic Laboratory, Logan, UT) using an inductively coupled argon plasma emission spectrometry method. Selenium was measured using an ICP-MS (ELAN 6000, Perkin Elmer, Shelton, CT) method as previously described [3].

### Nasal microbiota sample collection, 16s amplicon preparation, sequencing, and processing

During week 9 of the feeding trial, nasal swabs were collected from 5 or 6 weaned beef calves in each of the three treatment groups, by random selection of calves. One or two calves were selected from each pen (3 pens of 5 calves each per treatment group). Sterile, individually wrapped, polyester tipped applicators (Puritan^®^, Guilford, ME) were inserted approximately 10 cm into the nares, twirled to collect a mucosal swab, and then placed into individual sterile containers (10 mL, red topped, BD Vacutainer collection tubes; Becton Dickinson, Franklin Lakes, NJ) avoiding any contamination or contact with the wooden stick. Swabs in tubes were subsequently frozen at -80°C within 4 hours of collection. Negative control (sterile) swabs were similarly processed.

Microbial DNA was extracted from nasal swab samples using MoBio Power soil DNA isolation kit (MoBio Laboratories, USA) as per the manufacturer’s instructions. The V4 region of the 16S rRNA gene was amplified with primers 515F (5′-GTGCCAGCMGCCGCGGTAA-3′) and 806R (5′-GGACTACVSGGGTATCTAAT-3′) at the MR DNA Laboratory (Shallowater, TX, USA) and sequenced on an Illumina MiSeq instrument at the MR DNA Laboratory (Shallowater, TX, USA). Raw sequence data was screened, trimmed, filtered, denoised and barcodes and chimera sequences were depleted from the dataset using QIIME v1.8 [20] pipeline and UCHIME [21]. Operational taxonomic units (OTUs) were assigned based on at least 97% sequence similarity against the Greengenes reference database. For downstream analysis, sequences assigned as chloroplast, and mitochondria were removed. Sequences were rarefied to an even depth of 7,214 sequences per sample to account for unequal sequencing depth across samples. The sequences were deposited in NCBI SRA under accession number SRP090121.

### Statistical analysis

Statistical analyses were performed using SAS version 9.2 [22]. Whole-blood Se concentrations were analyzed as repeated-measures-in-time using PROC MIXED. Fixed effects in the model were Se application rate (0, 45.0, and 89.9 g Se/ha), gender, baseline WB-Se (as linear covariate), time (after 2, 4, 6, and 8 weeks of feeding Se enriched hay), and the interaction between Se application rate and time. An unstructured variance-covariance matrix was used to account for variation of measures within calves. The unstructured variance-covariance matrix provided the most parsimonious variance-covariance matrix based on the lowest value by the Aikaike Information Criterion. To evaluate the effect of Se application rate, linear and quadratic contrasts were constructed. In addition, the linear response of the dependent variable Se forage content or WB-Se concentrations of beef calves to the independent variable Se fertilization rate were evaluated using univariate regression in PROC REG. Mean values were used for each pen, as pen was the experimental unit. Data are reported as least square means ± SEM. Statistical significance was declared at *P* ≤ 0.05 and a tendency at 0.05 < *P* ≤ 0.10.

Nasal swabs were collected randomly from 5 or 6 calves in each of the three treatment groups and treated as individual samples. Differences in bacterial communities between the three groups were determined using the phylogeny based unweighted UniFrac distance metric and PCoA plots were generated with QIIME. ANOSIM (Analysis of Similarity) test within PRIMER 6 software package (PRIMER-E Ltd., Luton, UK) was performed on the weighted and unweighted UniFrac distance metrics to find significant differences in microbial communities between the groups.

All the datasets were tested for normality with Shapiro-Wilk test (JMP Pro 11, SAS Software Inc.). Since most of the datasets did not meet the assumptions of normal distribution, non-parametric Kruskal-Wallis tests were performed. The resulting *P*-values were then adjusted for multiple comparisons using the Benjamini & Hochberg’s False Discovery Rate (FDR) for each taxonomic level, and an adjusted *P* < 0.05 was considered statistically significant (Benjamini and Hochberg, 1995). A Dunn’s post-test was used to determine which diets were significantly different. A significance value of *P* < 0.05 was selected for all statistical tests. Linear discriminant analysis effect size (LEfSe) was utilized to identify bacterial taxa that were differentially abundant between the healthy controls and calves fed Se enriched alfalfa hay.

### PICRUSt

To determine if there were any changes in microbial function in the nasal microbiota, PICRUSt (Phylogenetic Investigation of Communities by Reconstruction of Unobserved States) was applied to make functional gene content predictions on the 16S rRNA gene sequencing data [23]. The results obtained with PICRUSt were analyzed using LEfSe to identify the significantly altered microbial functions between the groups.

## Results

### Agronomic biofortification

Fertilizing fields with increasing amounts of Na selenate increased the Se-concentration of third cutting alfalfa hay from 0.34 mg Se/kg dry matter (non-fertilized control) to 2.42 and 5.17 mg Se/kg dry matter for Na selenate application rates of 45.0 and 89.9 g Se/ha, respectively. The relationship between amount of Se applied by fertilization (g Se/ha) and observed forage Se concentration (mg Se/kg dry matter) was y = 0.054x + 0.228, *R*^2^ = 0.9935. Calculated Se intake from dietary sources was 3.0, 15.6, and 32.2 mg Se/head/day for calves consuming alfalfa hay with Se concentrations of 0.34, 2.42, and 5.17 mg Se/kg dry matter, respectively.

### Whole-blood selenium concentrations

**Fig 1:**
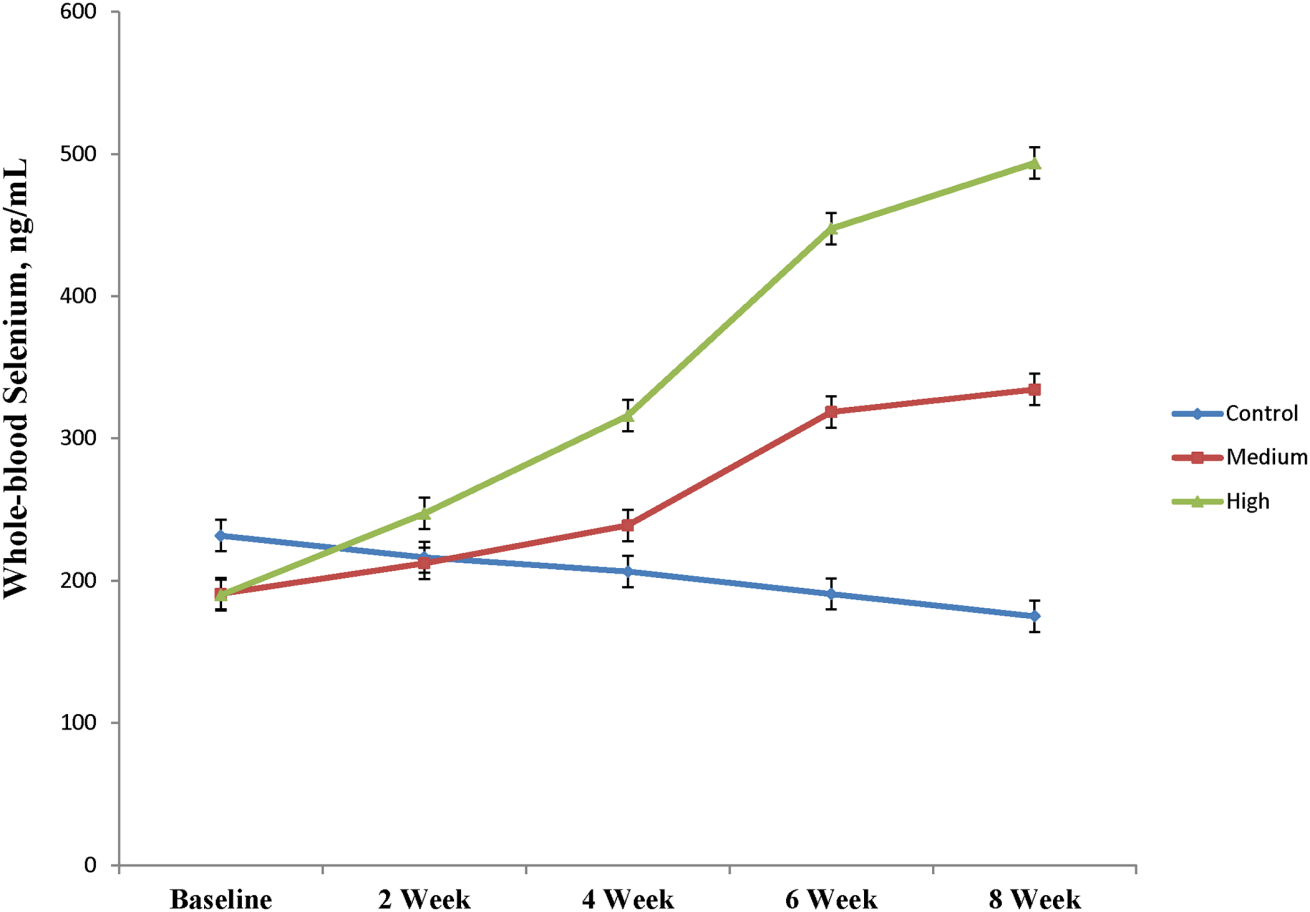
Whole-blood Se concentrations (mean ± SEM) for weaned beef calves that were consuming alfalfa hay harvested from fields with no Se fertilization (control; n = 3 pens with 5 calves/pen) or from fields fertilized with sodium-selenate at application rates of 45.0 (medium; n = 3 pens with 5 calves/pen) or 89.9 (high; n = 3 pens with 5 calves/pen) g Se/ha for 8 weeks. The normal reference interval for WB-Se concentrations of beef cattle is 120 to 300 ng/mL.

Prior to agronomic biofortification, calves had WB-Se concentrations (mean, 204 ng/mL; range, 42–307 ng/mL), which were mostly within the reference interval of adult cows (120–300 ng/mL). Whole blood-Se concentrations after 8 weeks of feeding Se-fertilized alfalfa hay were dependent upon Se-application rates (0, 45.0, or 89.9 g Se/ha) and were 175 ± 11, 335 ± 11, and 494 ± 11 ng/mL (Fig 1; *P*_Linear_ < 0.0001).

### Sequence analysis and alpha rarefaction

**Fig 2:**
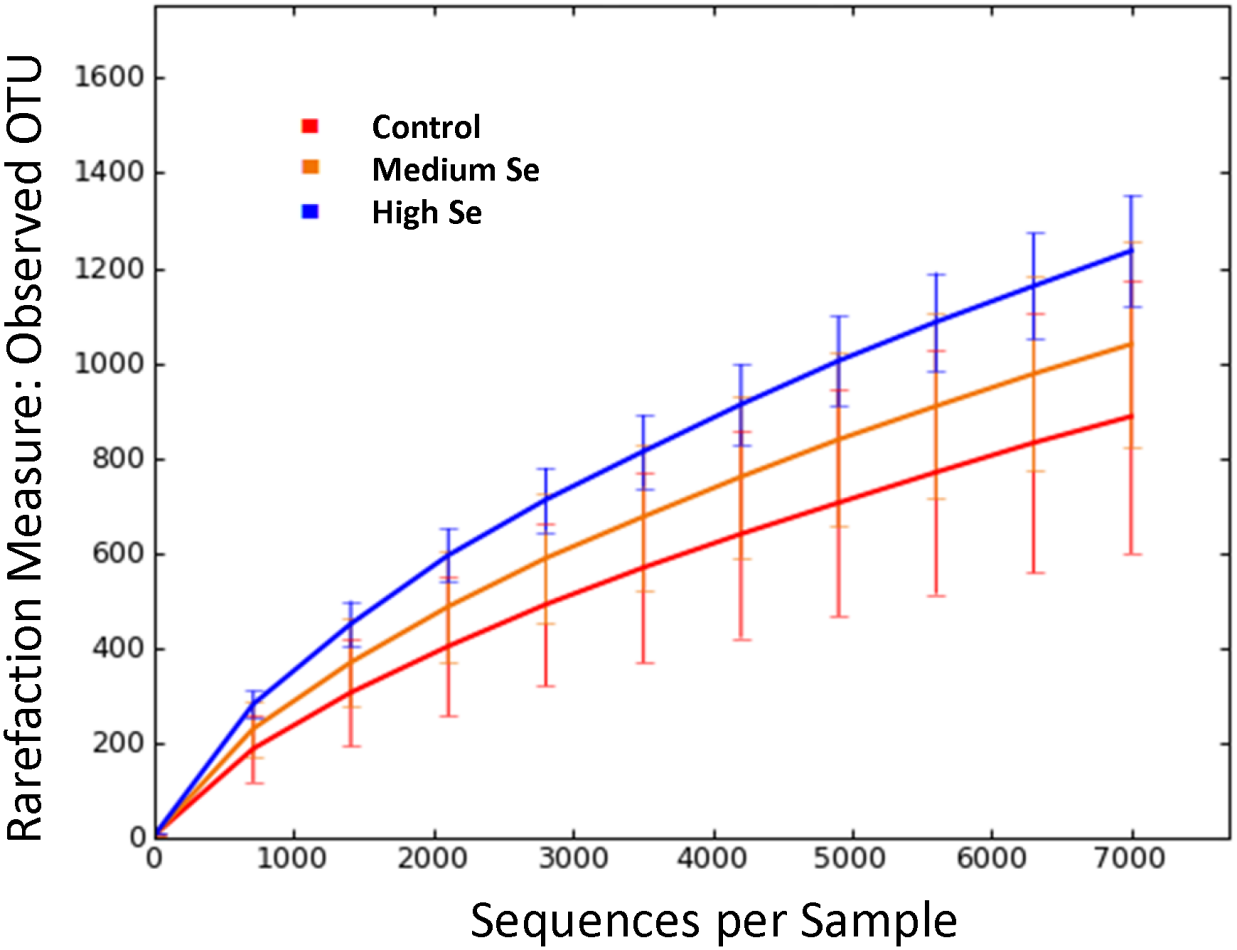
Rarefaction analysis of 16S rRNA gene sequences obtained from nasal samples of randomly chosen healthy control calves (n = 5) and calves fed either medium (n = 6) or high (n = 5) Se-enriched alfalfa hay for 8 weeks. Alfalfa hay was harvested from fields with no Se fertilization (control) or from fields fertilized with sodium-selenate at application rates of 45.0 (medium) or 89.9 (high) g Se/ha.

The sequence analysis yielded 1,137,749 quality sequences for all analyzed samples (n = 16; mean ± SD, 70,859 ± 10,178). Rarefaction analysis was performed at a depth of 7,214 sequences. Fig 2 shows the alpha rarefaction curve comparing species richness (observed OTUs) between weaned beef calves fed Se-biofortified alfalfa hay and healthy control calves. Alpha diversity as described by Chao1, Observed OTU and Shannon index were increased in calves fed Se-biofortified hay, although the increase was not statistically significant (Table 2; *P* > 0.1).

**Table 2:** Summary of alpha diversity measures per dietary Se treatment group^1^.

	Control (Low-Se)	Med-Se	High-Se	*P*-value
Chao1 (mean ± SD)	2123.1 ± 557.4	2253.7 ± 405.2	2611.6 ± 423.4	0.1838
Observed OTU (mean ± SD)	887.4 ± 322.3	997.6 ± 241.9	1236.0 ± 131.0	0.1439
Shannon Index (mean ± SD)	5.9 ± 1.3	6.2 ± 1.2	7.4 ± 0.7	0.1259

^1^Control calves consumed alfalfa hay harvested from fields with no Se fertilization (n = 5); Med-Se calves consumed alfalfa hay from fields fertilized with sodium-selenate at application rates of 45.0 g Se/ha for 8 weeks (n = 6); High-Se calves consumed alfalfa hay from fields fertilized with 89.9 g Se/ha for 8 weeks (n = 5)

### Microbial communities

**Fig 3:**
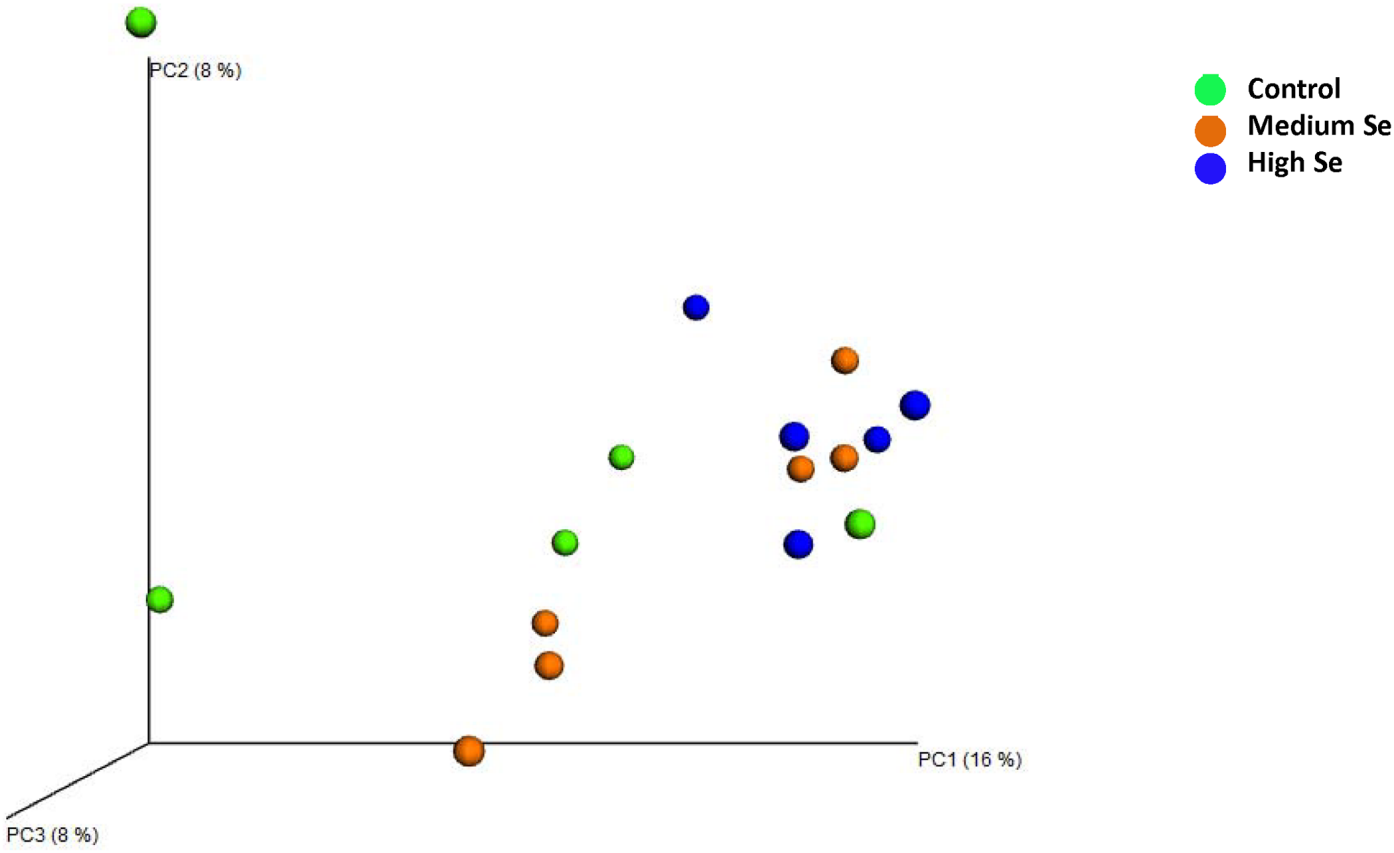
Principal coordinate analysis (PCoA) of unweighted UniFrac distances of 16S rRNA genes. Data obtained from nasal samples of randomly chosen healthy control calves (n = 5) and calves fed either medium (n = 6) or high (n = 5) Se-enriched alfalfa hay for 8 weeks. Alfalfa hay was harvested from fields with no Se fertilization (control) or from fields fertilized with sodium-selenate at application rates of 45.0 (medium) or 89.9 (high) g Se/ha.

Calves fed high Se-biofortified alfalfa hay clustered separately when compared with healthy control calves in the PCoA plot (Fig 3). ANOSIM of unweighted UniFrac distances showed that this clustering was significant (*R* = 0.362, *P* = 0.03). When analyzed separately, there was a significant difference in microbial communities between healthy control calves and calves fed high-Se alfalfa hay (*R* = 0.216, *P* = 0.04), however the clustering was not significant (*R* = 0.155, *P* = 0.093) between healthy control calves and calves fed medium-Se alfalfa hay (Table 3).

**Table 3:** ANOSIM values based on unweighted and weighted UniFrac distances for dietary Se treatment group comparisons^1^.

	*R*	*P* value
**Unweighted**		
Control, Med-Se	0.155	0.093
Control, High-Se	0.216	0.04
Med-Se, High-Se	0.067	0.225
**Weighted**		
Control, Med-Se	0.240	0.069
Control, High-Se	0.332	0.056
Med-Se, High-Se	-0.136	0.907

^1^Control calves consumed alfalfa hay harvested from fields with no Se fertilization (n = 5); Med-Se calves consumed alfalfa hay from fields fertilized with sodium-selenate at application rates of 45.0 g Se/ha for 8 weeks (n = 6); High-Se calves consumed alfalfa hay from fields fertilized with 89.9 g Se/ha for 8 weeks (n = 5)

**Fig 4:**
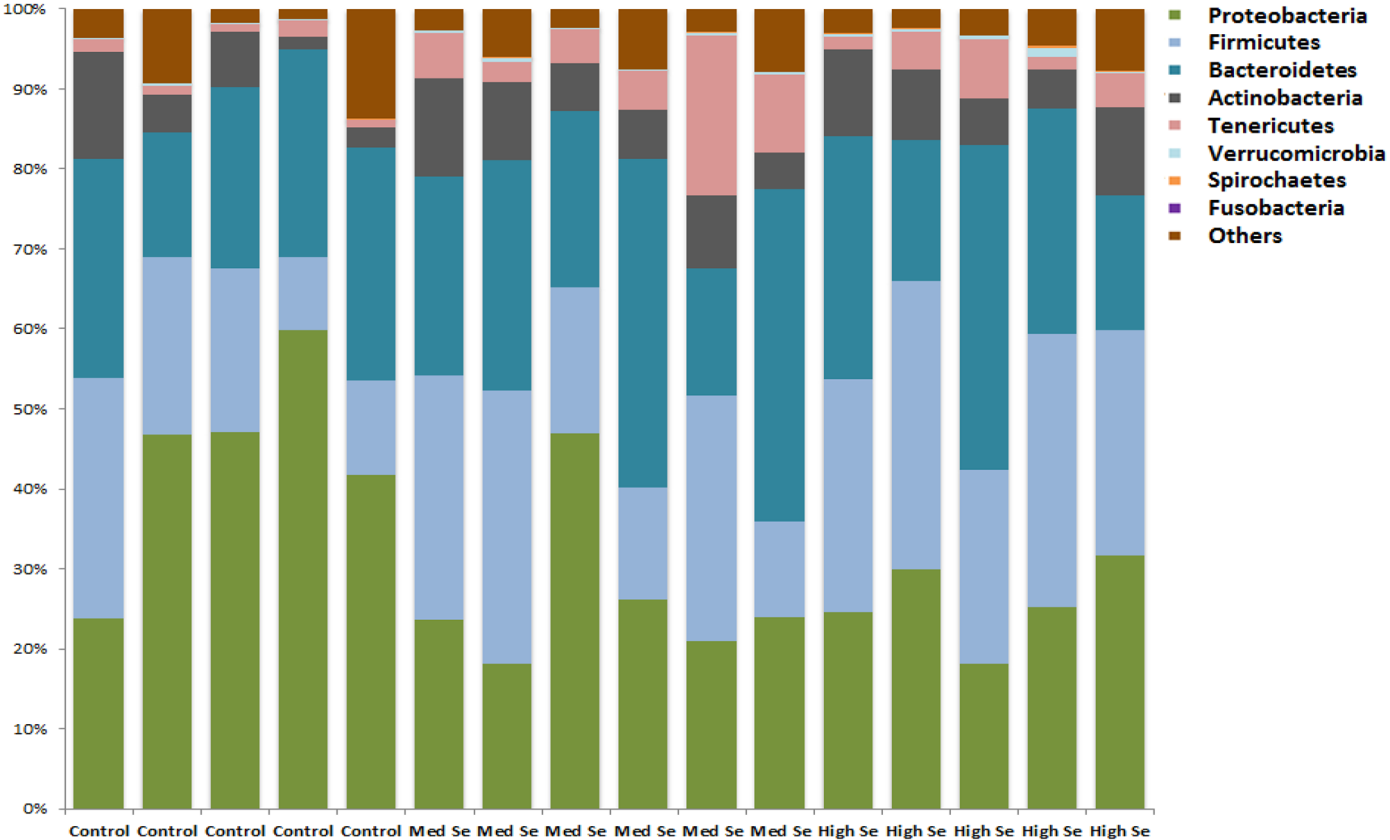
Relative abundance of the most prevalent bacterial phyla identified in the nasal samples of randomly chosen healthy control calves (n = 5) and calves fed either medium (n = 6) or high (n = 5) Se-enriched alfalfa hay for 8 weeks. Alfalfa hay was harvested from fields with no Se fertilization (control) or from fields fertilized with sodium-selenate at application rates of 45.0 (medium) or 89.9 (high) g Se/ha.

Relative abundances of the most abundant bacterial phyla in all three groups are shown in Fig 4. Based on LEfSe, a few bacterial groups were altered between the three groups (Table 4). The bacterial orders *Lactobacillales* and *Flavobacteriales* were increased in healthy controls. Meanwhile, *Clostridiales* and *Bacteroidales* were increased in calves fed with high Se alfalfa hay. However, when individual bacterial groups were compared between the three diet groups with Kruskal Wallis test and adjusted for multiple comparisons (S1 Table), there was no significant difference in any of the bacterial taxa.

**Table 4:** Linear discriminant analysis of bacterial taxa and their associations with dietary Se treatment^1^. Only a LDA score of >3 is shown.

	Diet	LDA
k__Bacteria|p__Proteobacteria|c__Gammaproteobacteria|o__Pasteurellales|f__Pasteurellaceae|g__Mannheimia	Med-Se	3.04
k__Bacteria|p__Proteobacteria|c__Gammaproteobacteria|o__Oceanospirillales|f__Halomonadaceae|g__CandidatusPortiera	High-Se	3.14
k__Bacteria|p__Bacteroidetes|c__Flavobacteriia|o__Flavobacteriales|f__Cryomorphaceae|g__	Med-Se	3.19
k__Bacteria|p__Proteobacteria|c__Betaproteobacteria|o__Burkholderiales|f__Alcaligenaceae|g__	Med-Se	3.25
k__Bacteria|p__Verrucomicrobia	High-Se	3.26
k__Bacteria|p__Firmicutes|c__Clostridia|o__Clostridiales|f__Lachnospiraceae|g__	High-Se	3.36
k__Bacteria|p__Firmicutes|c__Bacilli|o__Bacillales|f__Planococcaceae|g__Sporosarcina	Med-Se	3.40
k__Bacteria|p__Firmicutes|c__Clostridia|o__Clostridiales|f__Lachnospiraceae	High-Se	3.57
k__Bacteria|p__Bacteroidetes|c__Bacteroidia|o__Bacteroidales|f__Rikenellaceae	High-Se	3.63
k__Bacteria|p__Bacteroidetes|c__Bacteroidia|o__Bacteroidales|f__Rikenellaceae|g__	High-Se	3.63
k__Bacteria|p__Bacteroidetes|c__Bacteroidia|o__Bacteroidales|f__Bacteroidaceae|g__5_7N15	High-Se	3.70
k__Bacteria|p__Firmicutes|c__Bacilli|o__Lactobacillales	Control	4.03
k__Bacteria|p__Firmicutes|c__Bacilli|o__Lactobacillales|f__Carnobacteriaceae|g__Trichococcus	Control	4.04
k__Bacteria|p__Firmicutes|c__Bacilli|o__Lactobacillales|f__Carnobacteriaceae	Control	4.04
k__Bacteria|p__Tenericutes|c__Mollicutes	Med-Se	4.51
k__Bacteria|p__Tenericutes	Med-Se	4.52
k__Bacteria|p__Bacteroidetes|c__Flavobacteriia|o__Flavobacteriales|f___Weeksellaceae|g__	Control	4.53
k__Bacteria|p__Bacteroidetes|c__Flavobacteriia|o__Flavobacteriales|f___Weeksellaceae_	Control	4.57

^1^Control calves consumed alfalfa hay harvested from fields with no Se fertilization (n = 5); Med-Se calves consumed alfalfa hay from fields fertilized with sodium-selenate at application rates of 45.0 g Se/ha for 8 weeks (n = 6); High-Se calves consumed alfalfa hay from fields fertilized with 89.9 g Se/ha for 8 weeks (n = 5)

### Predicted functional composition of nasal microbial communities with PICRUSt

A total of 8 differentially abundant bacterial functions were observed between the three groups after LEfSe analysis (LDA score > 2.5) of the PICRUSt data (Table 5). Metabolic pathways related to fatty acid biosynthesis and peptidoglycan biosynthesis was enriched in the healthy controls while transcription machinery, glycolysis and gluconeogenesis was enriched in medium Se biofortified group and bacterial chemotaxis, transcription, sporulation, starch and sucrose metabolism was enriched in high Se biofortified group.

**Table 5:** Linear discriminant analysis of KEGG pathways encoded by the nasal microbiota and their associations with dietary Se treatment^1^. Only a LDA score of >2.5 is shown.

	Diet	LDA
Metabolism|LipidMetabolism|Fatty acid biosynthesis	Control	2.52
Metabolism|GlycanBiosynthesisandMetabolism|Peptidoglycan biosynthesis	Control	2.60
Metabolism|CarbohydrateMetabolism|Starch and sucrose metabolism	High-Se	2.59
GeneticInformationProcessing|Transcription	High-Se	2.87
Unclassified|CellularProcessesandSignaling|Sporulation	High-Se	2.91
CellularProcesses|CellMotility|Bacterial chemotaxis	High-Se	2.97
Metabolism|CarbohydrateMetabolism|Glycolysis_Gluconeogenesis	Med-Se	2.57
GeneticInformationProcessing|Transcription|Transcription machinery	Med-Se	2.71

^1^Control calves consumed alfalfa hay harvested from fields with no Se fertilization (n = 5); Med-Se calves consumed alfalfa hay from fields fertilized with sodium-selenate at application rates of 45.0 g Se/ha for 8 weeks (n = 6); High-Se calves consumed alfalfa hay from fields fertilized with 89.9 g Se/ha for 8 weeks (n = 5)

## Discussion

Bovine respiratory disease complex, also known as shipping fever, is one of the most common causes of morbidity and mortality in cattle entering into feedlots [11]. The nasopharygeal microbiota in cattle plays an important role in overall respiratory health, particularly when stressors such as weaning, transportation, and feed adaptation affect the normal respiratory defenses (reviewed in [24]). Recent evidence suggests that limiting colonization of opportunistic bacterial pathogens will reduce BRD in feedlot cattle (reviewed in [24]). However, vaccinations do not completely protect against infections. And, the use of low dosages of oral prophylactic and therapeutic group medications converts opportunistic pathogenic bacteria in the respiratory tract into reservoirs of antimicrobial resistance [25]. In one study [11], cattle diagnosed with BRD had significantly less bacterial diversity and fewer OTUs at feedlot entry prompting researchers to conclude that reduced diversity of nasopharyngeal microbiota may be a risk factor to BRD development. As a result, new strategies for preventing respiratory disease in feedlot cattle have been suggested, for example, nasal probiotics [24]. Our results suggest that feeding Se-biofortified alfalfa hay to weaned beef calves prior to entering the feedlot is another strategy for increasing nasopharyngeal microbial diversity.

Rarefaction curves comparing the species richness (observed OTU number) and overall diversity (Chao1, Observed OTU, and Shannon index) among weaned beef calves fed Se-biofortified alfalfa hay compared with healthy control calves showed that calves fed the highest concentration of Se-biofortified alfalfa hay had more (1236 vs 887) OTUs identified at a depth of 7,214 sequences. This number of OTUs was comparable with results previously reported in feedlot cattle [26]. Calves receiving Se-supplementation had linearly increasing alpha diversity measures, although increases were not statistically significant. Thus, weaned beef calves fed Se-biofortified hay tended to have an enriched nasal microbiota.

In our study, a total of 16 bacterial phyla were identified compared with 8 in the previous report [26]. In the control calves, the majority of the sequences were classified as *Proteobacteria* (44%), but in calves receiving Se-biofortified hay *Firmicutes* and *Bacteroidetes* were slightly more or equally prevalent. *Actinobacteria* and *Tenericutes* were also present in lower percentages. Three phyla showed significant differences among calves, in that calves receiving Se-biofortified hay had greater percentages of *Tenericutes*, *Lentisphaerae*, and *Planctomycetes*. The phyla composition in our calves fed Se-biofortified hay was more consistent with that of calves in a previous study that had been in the feedlot for 60 days vs calves at feedlot entry [26].

The beta diversity was measured using the phylogeny-based unweighted and weighted UniFrac distances. ANOSIM of unweighted UniFrac distances showed that this clustering was significant (*R* = 0.362, *P* = 0.03). When the unweighted UniFrac distances for nasopharygeal microbiota in control calves were compared with calves fed high-Se alfalfa hay, the two groups were different (*P* = 0.04) from one another, although the associated *R*-value was modest (0.216). The *R*-value was higher (0.332) for the weighted UniFrac distance between these two groups and tended to be significant (*P* = 0.056). Calves fed the medium-Se alfalfa hay also tended to be different from control calves (*P* = 0.069). Holman et al. [26] also reported that calves at feedlot entry and after 60 days in the feedlot were different from one another based on beta diversity, thus indicating difference in microbial structure across time.

The linear discriminant analysis of bacterial taxa showed that bacterial orders were altered between the control calves and calves fed Se-fortified hay. The bacterial orders *Lactobacillales* and *Flavobacteriales* were increased in healthy control calves compared with *Clostridiales* and *Bacteroidales* being increased in calves fed Se-biofortified alfalfa hay. Although there were strong trends, no significant differences were noted for any of the bacterial taxa. Thus, it is likely that many bacterial traits coexisting together, rather than differences in specific types of bacteria, are responsible for overall changes in the ecosystem.

Analysis of bacterial functions induced by feeding Se-biofortified alfalfa hay revealed differences in metabolic pathways. For example in calves fed high levels of Se-biofortified hay there were enriched metabolic pathways involved in bacterial chemotaxis, transcription, sporulation, and starch and sucrose metabolism. The overall significance of these findings requires further study.

Calves likely have greater Se requirements during periods of stress such as during weaning and movement to a feedlot, which is one of the most stressful times for beef calves [3]. Selenium plays an important role in immune responses of cattle. For example, in a previous study we showed that calves fed Se-biofortified alfalfa hay had higher antibody titers after vaccination with *J-5 E*. *coli* bacterin, and that vaccine-specific antibody titers increased linearly with increasing Se content of the hay [27]. We have seen a similar effect on antibody responses to *J-5 E*. *coli* bacterin in adult beef cows grazing Se-fertilized forage [28]. In the previous study with weaned beef calves, overall health was improved as feedlot calves had lower mortality and greater slaughter weights. Neutrophil total antioxidant potential also tended to be greater in calves fed Se-biofortified hay, and neutrophil gene expression was altered [27]. For example, two genes involved in innate immune function (IL-8R and L-Selectin) were lower in calves fed the highest level of Se-biofortified alfalfa hay. Genes involved in regulation of thioredoxin levels were also affected by Se treatment. Thus, our previous studies suggest that Se-supplementation above currently recommended levels may have short- and long-term beneficial effects on immune function and cattle production [3, 27, 28]. In particular, the role of Se in altering gene expression may be relevant to alterations in bacterial communities, as noted for functional food amino acids (reviewed in [8, 9]).

Research in the last 10 years has demonstrated that the microbiota is important in priming and orchestrating innate and adaptive immune responses of the host (reviewed in [29]). Whether Se-induced changes in the microbiota underlie the changes we have seen in innate and adaptive immune responses in weaned beef calves in previous studies requires more extensive research.

In conclusion, feeding Se-enriched alfalfa hay to weaned beef calves for 8 weeks during a preconditioning period to prepare them for the feedlot resulted in enrichment of the nasal microbiome. Calves fed the high-Se alfalfa hay had more OTUs, and beta diversity was different compared with control calves. The bacterial orders *Lactobacillales* and *Flavobacteriales* were increased in healthy control calves compared with *Clostridiales* and *Bacteroidales* being increased in calves fed Se-biofortified alfalfa hay. The mechanism by which dietary Se alters the nasal microbiome was not determined. Future studies are needed to investigate pathways linking dietary Se enrichment to gene expression, immune responses, and mucosal bacterial communities. Feeding Se-biofortified alfalfa hay to weaned beef calves prior to entering the feedlot is a strategy for increasing nasopharyngeal microbial diversity, and may ameliorate a negative risk factor for BRD development.

## Supporting information

S1 Table Individual bacterial group percentages. Mean percentages of the most abundant bacterial groups on the various phylogenetic levels (phylum, class, order, family, genus) identified in nasal samples of randomly chosen healthy control calves (n = 5) and calves fed either medium (n = 6) or high (n = 5) Se-enriched alfalfa hay for 8 weeks. Alfalfa hay was harvested from fields with no Se fertilization (control) or from fields fertilized with sodium-selenate at application rates of 45.0 (medium) or 89.9 (high) g Se/ha.
(DOCX)

## Abbreviations

ADF: acid detergent fiber
ANOSIM: analysis of similarity
BRD: bovine respiratory disease
CP: crude protein
FDR: false discovery rate
ICP-MS: inductively coupled argon plasma emission spectroscopy
LDA: linear discriminant analysis
LEfSe: linear discriminant analysis effect size
NDF: neutral detergent fiber
OTU: operational taxonomic units
PICRUSt: phylogenetic investigation of communities by reconstruction of unobserved states
ROS: reactive oxygen species
Se: selenium
WB-Se: whole blood-selenium

## References

[1] Fairweather-Tait SJ, Collings R, Hurst R. Selenium bioavailability: Current knowledge and future research requirements. Am J Clin Nutr. 2010;91(5):1484S–91S. Epub 2010/03/05. 10.3945/ajcn.2010.28674J . 20200264

[2] Hugejiletu H, Bobe G, Vorachek WR, Gorman ME, Mosher WD, Pirelli GJ, et al Selenium supplementation alters gene expression profiles associated with innate immunity in whole-blood neutrophils of sheep. Biol Trace Elem Res. 2013;154(1):28–44. Epub 2013/06/12. 10.1007/s12011-013-9716-6 . 23754590

[3] Hall JA, Bobe G, Hunter JK, Vorachek WR, Stewart WC, Vanegas JA, et al Effect of feeding selenium-fertilized alfalfa hay on performance of weaned beef calves. PLoS One. 2013;8(3):e58188 Epub 2013/03/29. 10.1371/journal.pone.0058188 ; 23536788 PMC3594272

[4] Hall JA, Van Saun RJ, Nichols T, Mosher W, Pirelli G. Comparison of selenium status in sheep after short-term exposure to high-selenium-fertilized forage or mineral supplement. Small Ruminant Res. 2009;82(1):40–5. 10.1016/j.smallrumres.2009.01.010

[5] Hall JA, Van Saun RJ, Bobe G, Stewart WC, Vorachek WR, Mosher WD, et al Organic and inorganic selenium: I. Oral bioavailability in ewes. J Anim Sci. 2012;90(2):568–76. Epub 2011/10/04. 10.2527/jas.2011-4075 . 21965451

[6] O'Toole D, Raisbeck MF. Pathology of experimentally induced chronic selenosis (alkali disease) in yearling cattle. J Vet Diagn Invest. 1995;7(3):364–73. Epub 1995/07/01. 10.1177/104063879500700312 . 7578453

[7] Whanger PD. Selenocompounds in plants and animals and their biological significance. J Am Coll Nutr. 2002;21(3):223–32. Epub 2002/06/21. . 12074249 10.1080/07315724.2002.10719214

[8] Wu M, Xiao H, Liu G, Chen S, Tan B, Ren W, et al Glutamine promotes intestinal sIgA secretion through intestinal microbiota and IL-13. Mol Nutr Food Res. 2016;60(7):1637–48. Epub 2016/03/24. 10.1002/mnfr.201600026 . 27005687

[9] Ren W, Wang K, Yin J, Chen S, Liu G, Tan B, et al Glutamine-induced secretion of intestinal secretory immunoglobulin A: A mechanistic perspective. Front Immunol. 2016;7:503 Epub 2016/12/10. 10.3389/fimmu.2016.00503 ; 27933057 PMC5121228

[10] de Steenhuijsen Piters WAA, Sanders EAM, Bogaert D. The role of the local microbial ecosystem in respiratory health and disease. Philos T R Soc B. 2015;370(1675). 10.1098/rstb.2014.0294 PMC4528492 26150660

[11] Holman DB, McAllister TA, Topp E, Wright ADG, Alexander TW. The nasopharyngeal microbiota of feedlot cattle that develop bovine respiratory disease. Vet Microbiol. 2015;180(1–2):90–5. 10.1016/j.vetmic.2015.07.031 . 26249828

[12] Timsit E, Workentine M, Schryvers AB, Holman DB, van der Meer F, Alexander TW. Evolution of the nasopharyngeal microbiota of beef cattle from weaning to 40 days after arrival at a feedlot. Vet Microbiol. 2016;187:75–81. 10.1016/j.vetmic.2016.03.020 . 27066712

[13] Pelton SI. Regulation of bacterial trafficking in the nasopharynx. Paediatr Respir Rev. 2012;13(3):150–3. 10.1016/j.prrv.2012.04.001 . 22726870 PMC3383606

[14] MWPS-6. Beef Housing and Equipment Handbook. 4th ed Iowa State University, Ames, IA, USA: Midwest Plan Service; 1987.

[15] NRC. Nutrient Requirements of Beef Cattle: 7th rev ed: Update 2000. Washington DC: National Academy Press, 2000.

[16] AOAC. Official Methods of Analysis. 17th ed Arlington, VA, USA: Association of Official Analytical Chemists; 2000.

[17] Van Soest PJ, Robertson JB, Lewis BA. Methods for dietary fiber, neutral detergent fiber, and nonstarch polysaccharides in relation to animal nutrition. J Dairy Sci. 1991;74(10):3583–97. Epub 1991/10/01. 10.3168/jds.S0022-0302(91)78551-2 . 1660498

[18] Krishnamoorthy U, Muscato TV, Sniffen CJ, Vansoest PJ. Nitrogen fractions in selected feedstuffs. J Dairy Sci. 1982;65(2):217–25.

[19] Davis TZ, Stegelmeier BL, Panter KE, Cook D, Gardner DR, Hall JO. Toxicokinetics and pathology of plant-associated acute selenium toxicosis in steers. J Vet Diagn Invest. 2012;24(2):319–27. 10.1177/1040638711435407 . 22379047

[20] Caporaso JG, Kuczynski J, Stombaugh J, Bittinger K, Bushman FD, Costello EK, et al QIIME allows analysis of high-throughput community sequencing data. Nat Methods. 2010;7(5):335–6. 10.1038/nmeth.f.303 ; 20383131 PMC3156573

[21] Edgar RC, Haas BJ, Clemente JC, Quince C, Knight R. UCHIME improves sensitivity and speed of chimera detection. Bioinformatics. 2011;27(16):2194–200. 10.1093/bioinformatics/btr381 ; 21700674 PMC3150044

[22] SAS Institute. SAS User’s Guide. Statistics, Version 9.2 Cary, NC: SAS Inst Inc; 2009.

[23] Langille MGI, Zaneveld J, Caporaso JG, McDonald D, Knights D, Reyes JA, et al Predictive functional profiling of microbial communities using 16S rRNA marker gene sequences. Nat Biotechnol. 2013;31(9):814–+. 10.1038/nbt.2676 23975157 PMC3819121

[24] Timsit E, Holman DB, Hallewell J, Alexander TW. The nasopharyngeal microbiota in feedlot cattle and its role in respiratory health. Animal Frontiers. 2016;6(2):44–50. 10.2527/af.2016-0022

[25] Catry B, Dewulf J, Maes D, Pardon B, Callens B, Vanrobaeys M, et al Effect of antimicrobial consumption and production type on antibacterial resistance in the bovine respiratory and digestive tract. PLoS One. 2016;11(1):e0146488 Epub 2016/01/29. 10.1371/journal.pone.0146488 ; 26820134 PMC4731056

[26] Holman DB, Timsit E, Alexander TW. The nasopharyngeal microbiota of feedlot cattle. Sci Rep. 2015;5:15557 Epub 2015/10/27. 10.1038/srep15557 ; 26497574 PMC4620444

[27] Hall JA, Bobe G, Vorachek WR, Hugejiletu, Gorman ME, Mosher WD, et al Effects of feeding selenium-enriched alfalfa hay on immunity and health of weaned beef calves. Biol Trace Elem Res. 2013;156(1–3):96–110. Epub 2013/10/22. 10.1007/s12011-013-9843-0 . 24142411

[28] Hall JA, Harwell AM, Van Saun RJ, Vorachek WR, Stewart WC, Galbraith ML, et al Agronomic biofortification with selenium: Effects on whole blood selenium and humoral immunity in beef cattle. Anim Feed Sci Tech. 2011;164(3–4):184–90. 10.1016/j.anifeedsci.2011.01.009

[29] Gunther C, Josenhans C, Wehkamp J. Crosstalk between microbiota, pathogens and the innate immune responses. Int J Med Microbiol. 2016;306(5):257–65. 10.1016/j.ijmm.2016.03.003 . 26996809

