# Supplementary material for: Weaned beef calves fed selenium-biofortified alfalfa hay have an enriched nasal microbiota compared with healthy controls
Source: PLoS One. 2017 Jun 8;12(6):e0179215. doi: 10.1371/journal.pone.0179215 (PMC5464631; doi:10.1371/journal.pone.0179215)
Supplement: S1 Table — Mean percentages of the most abundant bacterial groups on the various phylogenetic levels (phylum, class, order, family, genus) identified in nasal samples of randomly chosen healthy control calves (n = 5) and calves fed either medium (n = 6) or high (n = 5) Se-enriched alfalfa hay for 8 weeks. Alfalfa hay was harvested from fields with no Se fertilization (control) or from fields fertilized with sodium-selenate at application rates of 45.0 (medium) or 89.9 (high) g Se/ha. (DOCX) [file pone.0179215.s001.docx]

**S1 Table. Individual bacterial group percentages.** Mean percentages of the most abundant bacterial groups on the various phylogenetic levels (phylum, class, order, family, genus) identified in nasal samples of randomly chosen healthy control calves (n=5) and calves fed either medium (n=6) or high (n=5) Se-enriched alfalfa hay for 8 weeks. Alfalfa hay was harvested from fields with no Se fertilization (control) or from fields fertilized with sodium-selenate at application rates of 45.0 (medium) or 89.9 (high) g Se/ha.

|  | **Mean (%)** | | |  | **SD (%)^1^** | | | |  |
| --- | --- | --- | --- | --- | --- | --- | --- | --- | --- |
|  | Control | Med-Se | High- Se |  | Control | Med-Se | High-Se | *P*-value | *P*-value^2^ |
|  |  |  |  |  |  |  |  |  |  |
| **Phylum** |  |  |  |  |  |  |  |  |  |
| Acidobacteria | 0 | 0.01 | 0.01 |  | 0.01 | 0.01 | 0.01 | 0.571 | 0.790 |
| Actinobacteria | 6.07 | 7.66 | 7.62 |  | 5.07 | 2.72 | 2.75 | 0.615 | 0.790 |
| Bacteroidetes | 23.88 | 29.37 | 29.25 |  | 5.02 | 10.38 | 9.24 | 0.534 | 0.790 |
| Chloroflexi | 0.07 | 0.05 | 0.05 |  | 0.09 | 0.06 | 0.03 | 0.889 | 0.889 |
| Cyanobacteria | 1.27 | 0.7 | 0.27 |  | 1.78 | 1.12 | 0.18 | 0.885 | 0.889 |
| Elusimicrobia | 0.01 | 0.01 | 0.01 |  | 0.01 | 0.01 | 0.01 | 0.385 | 0.630 |
| Firmicutes | 18.86 | 23.28 | 30.80 |  | 8.85 | 9.16 | 5.33 | 0.140 | 0.465 |
| GN02 | 1.30 | 0.06 | 0.04 |  | 2.73 | 0.06 | 0.08 | 0.345 | 0.630 |
| Lentisphaerae | 0.2^a^ | 0.3^a,b^ | 0.51^b^ |  | 0.11 | 0.24 | 0.27 | 0.038 | 0.225 |
| Planctomycetes | 0.01^a^ | 0.01^a,b^ | 0.03^b^ |  | 0.01 | 0.01 | 0.01 | 0.026 | 0.225 |
| Proteobacteria | 43.61 | 26.97 | 24.50 |  | 13.23 | 10.54 | 4.31 | 0.183 | 0.465 |
| Spirochaetes | 0.05 | 0.04 | 0.11 |  | 0.04 | 0.03 | 0.06 | 0.105 | 0.465 |
| TM7 | 0.01 | 0.00 | 0.00 |  | 0.01 | 0.01 | 0.01 | 0.207 | 0.465 |
| Tenericutes | 1.37^a^ | 7.64^a,b^ | 4.14^a,b^ |  | 0.59 | 6.18 | 2.69 | 0.011 | 0.198 |
| Verrucomicrobia | 0.17 | 0.22 | 0.45 |  | 0.13 | 0.13 | 0.27 | 0.159 | 0.465 |
| [Thermi] | 1.12 | 0.04 | 0.04 |  | 2.42 | 0.02 | 0.02 | 0.848 | 0.889 |
|  |  |  |  |  |  |  |  |  |  |
| **Class** |  |  |  |  |  |  |  |  |  |
| Solibacteres | 0.00 | 0.0 | 0.0 |  | 0 | 0.01 | 0 | 0.072 | 0.298 |
| Acidimicrobiia | 0.01 | 0.0 | 0.0 |  | 0.01 | 0.03 | 0.02 | 0.319 | 0.593 |
| Actinobacteria | 5.97 | 7.5 | 7.5 |  | 4.99 | 2.64 | 2.76 | 0.615 | 0.789 |
| Coriobacteriia | 0.08 | 0.1 | 0.1 |  | 0.07 | 0.06 | 0.04 | 0.623 | 0.789 |
| Thermoleophilia | 0.01 | 0.0 | 0.0 |  | 0.01 | 0.01 | 0.01 | 0.036 | 0.231 |
| Bacteroidia | 2.82 | 4.4 | 8.2 |  | 2.14 | 2.66 | 3.05 | 0.065 | 0.298 |
| Cytophagia | 0.06 | 0.1 | 0.1 |  | 0.05 | 0.07 | 0.04 | 0.965 | 0.965 |
| Flavobacteriia | 7.45 | 0.4 | 0.6 |  | 8.61 | 0.06 | 0.4 | 0.086 | 0.317 |
| Sphingobacteriia | 0.76 | 0.8 | 1.1 |  | 0.74 | 0.26 | 0.28 | 0.328 | 0.593 |
| [Saprospirae] | 12.78 | 23.7 | 19.4 |  | 9.43 | 11.7 | 11.69 | 0.330 | 0.593 |
| Thermomicrobia | 0.07 | 0.0 | 0.0 |  | 0.09 | 0.06 | 0.04 | 0.863 | 0.887 |
| 4C0d-2 | 0.06 | 0.1 | 0.2 |  | 0.06 | 0.12 | 0.08 | 0.353 | 0.593 |
| Elusimicrobia | 0.01 | 0.0 | 0.0 |  | 0.01 | 0.01 | 0.01 | 0.385 | 0.594 |
| Bacilli | 12.36 | 13.1 | 14.1 |  | 4.33 | 3.62 | 3.61 | 0.725 | 0.813 |
| Clostridia | 6.42 | 10.1 | 16.5 |  | 4.62 | 5.6 | 4.86 | 0.110 | 0.340 |
| Erysipelotrichi | 0.08 | 0.1 | 0.2 |  | 0.05 | 0.09 | 0.1 | 0.597 | 0.789 |
| GN02;c__BD1-5 | 1.30 | 0.1 | 0.0 |  | 2.73 | 0.06 | 0.08 | 0.345 | 0.593 |
| [Lentisphaeria] | 0.17^a^ | 0.33^a,b^ | 0.51^b^ |  | 0.11 | 0.24 | 0.27 | 0.038 | 0.231 |
| Planctomycetia | 0.01^a^ | 0.01^a,b^ | 0.03^b^ |  | 0.01 | 0.01 | 0.01 | 0.026 | 0.231 |
| Alphaproteobacteria | 1.78 | 1.4 | 1.7 |  | 1.11 | 0.71 | 0.52 | 0.640 | 0.789 |
| Betaproteobacteria | 2.11 | 2.2 | 2.2 |  | 1.74 | 0.72 | 1.01 | 0.688 | 0.795 |
| Deltaproteobacteria | 0.37 | 0.45 | 0.38 |  | 0.51 | 0.48 | 0.13 | 0.375 | 0.594 |
| Epsilonproteobacteria | 0.06^a^ | 0.08^a,b^ | 0.17^b^ |  | 0.04 | 0.03 | 0.07 | 0.010 | 0.191 |
| Gammaproteobacteria | 39.29 | 22.77 | 20.06 |  | 15.16 | 11.32 | 3.99 | 0.225 | 0.521 |
| Spirochaetes | 0.05 | 0.04 | 0.11 |  | 0.04 | 0.03 | 0.06 | 0.101 | 0.340 |
| TM7;c__TM7-3 | 0.01 | 0 | 0 |  | 0.01 | 0.01 | 0.01 | 0.207 | 0.510 |
| Mollicutes | 1.30^a^ | 7.49^b^ | 3.91^a,b^ |  | 0.61 | 6.21 | 2.71 | 0.024 | 0.231 |
| RF3 | 0.07^a^ | 0.15^a,b^ | 0.23^b^ |  | 0.05 | 0.13 | 0.1 | 0.046 | 0.241 |
| Opitutae | 0.00 | 0.01 | 0.01 |  | 0.01 | 0.01 | 0.01 | 0.307 | 0.593 |
| Verruco-5 | 0.09 | 0.12 | 0.23 |  | 0.06 | 0.08 | 0.15 | 0.204 | 0.510 |
| Verrucomicrobiae | 0.08 | 0.09 | 0.21 |  | 0.08 | 0.06 | 0.12 | 0.137 | 0.390 |
| Deinococci | 1.12 | 0.04 | 0.04 |  | 2.42 | 0.02 | 0.02 | 0.848 | 0.887 |
|  |  |  |  |  |  |  |  |  |  |
| **Order** |  |  |  |  |  |  |  |  |  |
| Solibacterales | 0 | 0.01 | 0.00 |  | 0 | 0.01 | 0 | 0.072 | 0.286 |
| Acidimicrobiales | 0.01 | 0.03 | 0.01 |  | 0.01 | 0.03 | 0.02 | 0.319 | 0.45 |
| Actinomycetales | 5.96 | 7.50 | 7.46 |  | 4.97 | 2.59 | 2.75 | 0.615 | 0.693 |
| Bifidobacteriales | 0.01 | 0.04 | 0.01 |  | 0.02 | 0.07 | 0.01 | 0.799 | 0.826 |
| Coriobacteriales | 0.08 | 0.09 | 0.11 |  | 0.07 | 0.06 | 0.04 | 0.623 | 0.693 |
| Solirubrobacterales | 0.00a | 0.00^a,b^ | 0.02^b^ |  | 0.01 | 0.01 | 0.01 | 0.021 | 0.256 |
| Bacteroidales | 2.82 | 4.43 | 8.16 |  | 2.14 | 2.66 | 3.05 | 0.065 | 0.286 |
| Cytophagales | 0.06 | 0.07 | 0.06 |  | 0.05 | 0.07 | 0.04 | 0.965 | 0.919 |
| Flavobacteriales | 7.45 | 0.38 | 0.58 |  | 8.61 | 0.06 | 0.4 | 0.086 | 0.286 |
| Sphingobacteriales | 0.76 | 0.76 | 1.05 |  | 0.74 | 0.26 | 0.28 | 0.328 | 0.45 |
| [Saprospirales] | 12.78 | 23.73 | 19.40 |  | 9.43 | 11.7 | 11.69 | 0.330 | 0.45 |
| JG30-KF-CM45 | 0.07 | 0.04 | 0.04 |  | 0.09 | 0.06 | 0.04 | 0.863 | 0.849 |
| 4C0d-2;o__YS2 | 0.06 | 0.13 | 0.15 |  | 0.06 | 0.12 | 0.08 | 0.353 | 0.46 |
| Streptophyta | 1.2 | 0.57 | 0.12 |  | 1.77 | 1.03 | 0.1 | 0.613 | 0.693 |
| Elusimicrobiales | 0.01 | 0.01 | 0.01 |  | 0.01 | 0.01 | 0.01 | 0.385 | 0.492 |
| Bacillales | 7.09 | 9.99 | 10.54 |  | 4.09 | 3.22 | 2.32 | 0.305 | 0.449 |
| Lactobacillales | 5.17 | 3.04 | 3.46 |  | 1.12 | 0.95 | 1.42 | 0.045 | 0.286 |
| Turicibacterales | 0.1 | 0.05 | 0.12 |  | 0.09 | 0.03 | 0.09 | 0.299 | 0.449 |
| Clostridiales | 6.42 | 10.08 | 16.51 |  | 4.62 | 5.6 | 4.86 | 0.110 | 0.308 |
| Erysipelotrichales | 0.08 | 0.12 | 0.16 |  | 0.05 | 0.09 | 0.1 | 0.597 | 0.693 |
| c__BD1-5;o__ | 1.3 | 0.06 | 0.04 |  | 2.73 | 0.06 | 0.08 | 0.345 | 0.46 |
| Victivallales | 0.17^a^ | 0.33^ab^ | 0.51^b^ |  | 0.11 | 0.24 | 0.27 | 0.038 | 0.286 |
| Pirellulales | 0.01^ab^ | 0.01^b^ | 0.03^a^ |  | 0.01 | 0.01 | 0.01 | 0.020 | 0.256 |
| Alphaproteobacteria;o__ | 0.03 | 0.07 | 0.05 |  | 0.02 | 0.03 | 0.02 | 0.113 | 0.308 |
| BD7-3 | 0 | 0.01 | 0.00 |  | 0 | 0.01 | 0.01 | 0.196 | 0.379 |
| Caulobacterales | 0.03 | 0.04 | 0.08 |  | 0.02 | 0.03 | 0.02 | 0.065 | 0.286 |
| RF32 | 0.04 | 0.06 | 0.12 |  | 0.06 | 0.05 | 0.06 | 0.158 | 0.353 |
| Rhizobiales | 0.47 | 0.40 | 0.48 |  | 0.44 | 0.25 | 0.21 | 0.734 | 0.787 |
| Rhodobacterales | 0.35 | 0.42 | 0.57 |  | 0.32 | 0.22 | 0.32 | 0.521 | 0.638 |
| Rhodospirillales | 0.00^a^ | 0.00^ab^ | 0.01^b^ |  | 0 | 0 | 0.01 | 0.008 | 0.206 |
| Rickettsiales | 0.55 | 0.16 | 0.05 |  | 1.04 | 0.32 | 0.02 | 0.540 | 0.648 |
| Sphingomonadales | 0.3 | 0.29 | 0.36 |  | 0.21 | 0.19 | 0.06 | 0.286 | 0.449 |
| Burkholderiales | 1.14 | 2.08 | 2.04 |  | 0.86 | 0.67 | 0.99 | 0.111 | 0.308 |
| Neisseriales | 0.95 | 0.06 | 0.07 |  | 1.78 | 0.03 | 0.03 | 0.177 | 0.354 |
| Rhodocyclales | 0.02 | 0.07 | 0.05 |  | 0.02 | 0.04 | 0.04 | 0.055 | 0.286 |
| Bdellovibrionales | 0.03 | 0.02 | 0.03 |  | 0.04 | 0.02 | 0.02 | 0.821 | 0.835 |
| Desulfovibrionales | 0.33 | 0.42 | 0.35 |  | 0.49 | 0.48 | 0.13 | 0.302 | 0.449 |
| GMD14H09 | 0.01 | 0.01 | 0.00 |  | 0.02 | 0.01 | 0 | 0.168 | 0.353 |
| Campylobacterales | 0.06^a^ | 0.08^ab^ | 0.17^b^ |  | 0.04 | 0.03 | 0.07 | 0.010 | 0.206 |
| Aeromonadales | 0 | 0.00 | 0.01 |  | 0 | 0.01 | 0.01 | 0.171 | 0.353 |
| Alteromonadales | 1.66 | 2.59 | 3.30 |  | 1.44 | 0.6 | 1.39 | 0.150 | 0.353 |
| Cardiobacteriales | 0.36 | 0.21 | 0.09 |  | 0.42 | 0.23 | 0.1 | 0.209 | 0.379 |
| Enterobacteriales | 0.03 | 0.03 | 0.04 |  | 0.03 | 0.02 | 0.04 | 0.950 | 0.919 |
| Oceanospirillales | 0.33 | 0.60 | 0.54 |  | 0.15 | 0.27 | 0.6 | 0.307 | 0.449 |
| Pasteurellales | 10.74 | 2.30 | 2.68 |  | 11.56 | 2.58 | 2.58 | 0.306 | 0.449 |
| Pseudomonadales | 25.75 | 16.38 | 12.54 |  | 10.24 | 9.49 | 3.09 | 0.168 | 0.353 |
| Thiotrichales | 0.1 | 0.18 | 0.27 |  | 0.07 | 0.08 | 0.12 | 0.073 | 0.286 |
| Xanthomonadales | 0.31 | 0.46 | 0.60 |  | 0.36 | 0.39 | 0.2 | 0.149 | 0.353 |
| Spirochaetales | 0.05 | 0.04 | 0.11 |  | 0.04 | 0.03 | 0.06 | 0.085 | 0.286 |
| TM7-3;o__ | 0.01 | 0.00 | 0.00 |  | 0.01 | 0 | 0 | 0.030 | 0.286 |
| Acholeplasmatales | 0.48 | 1.37 | 1.30 |  | 0.39 | 0.7 | 1.38 | 0.082 | 0.286 |
| Anaeroplasmatales | 0.03 | 0.05 | 0.12 |  | 0.02 | 0.05 | 0.09 | 0.238 | 0.419 |
| Mycoplasmatales | 0.73 | 5.99 | 2.34 |  | 0.75 | 6.44 | 2.99 | 0.080 | 0.286 |
| RF39 | 0.05 | 0.08 | 0.15 |  | 0.06 | 0.06 | 0.06 | 0.091 | 0.288 |
| ML615J-28 | 0.07^a^ | 0.15^ab^ | 0.23^b^ |  | 0.05 | 0.13 | 0.1 | 0.046 | 0.286 |
| [Cerasicoccales] | 0 | 0.01 | 0.01 |  | 0 | 0.01 | 0.01 | 0.284 | 0.449 |
| WCHB1-41 | 0.09 | 0.12 | 0.23 |  | 0.06 | 0.08 | 0.15 | 0.204 | 0.379 |
| Verrucomicrobiales | 0.08 | 0.09 | 0.21 |  | 0.08 | 0.06 | 0.12 | 0.137 | 0.353 |
| Deinococcales | 1.12 | 0.04 | 0.04 |  | 2.42 | 0.02 | 0.02 | 0.848 | 0.848 |
|  |  |  |  |  |  |  |  |  |  |
| **Family** |  |  |  |  |  |  |  |  |  |
| Acidimicrobiales;f__ | 0.01 | 0.02 | 0.03 |  | 0.01 | 0.03 | 0.03 | 0.697 | 0.8 |
| Actinomycetales;Other | 0.02 | 0.01 | 0.02 |  | 0.01 | 0.01 | 0.01 | 0.087 | 0.431 |
| Actinomycetales;f__ | 0.01 | 0.02 | 0.02 |  | 0.02 | 0.02 | 0.01 | 0.594 | 0.741 |
| Actinomycetaceae | 0.06 | 0.05 | 0.08 |  | 0.06 | 0.03 | 0.06 | 0.769 | 0.855 |
| Bogoriellaceae | 0.05 | 0.03 | 0.05 |  | 0.07 | 0.04 | 0.02 | 0.564 | 0.724 |
| Brevibacteriaceae | 0.16 | 0.1 | 0.15 |  | 0.14 | 0.15 | 0.08 | 0.270 | 0.518 |
| Cellulomonadaceae | 0.1 | 0.07 | 0.09 |  | 0.13 | 0.05 | 0.07 | 0.990 | 0.992 |
| Corynebacteriaceae | 2.52 | 3.84 | 4.04 |  | 1.84 | 1.09 | 1.03 | 0.470 | 0.651 |
| Dermabacteraceae | 0.29 | 0.26 | 0.25 |  | 0.31 | 0.28 | 0.11 | 0.621 | 0.746 |
| Dermacoccaceae | 0.07 | 0.06 | 0.04 |  | 0.09 | 0.1 | 0.03 | 0.965 | 0.986 |
| Dietziaceae | 0.3 | 0.32 | 0.28 |  | 0.35 | 0.3 | 0.09 | 0.795 | 0.869 |
| Intrasporangiaceae | 0.21 | 0.18 | 0.2 |  | 0.2 | 0.1 | 0.14 | 0.753 | 0.845 |
| Jonesiaceae | 0 | 0.03 | 0.01 |  | 0.01 | 0.05 | 0.01 | 0.205 | 0.478 |
| Kineosporiaceae | 0.01 | 0 | 0.02 |  | 0.02 | 0.01 | 0.01 | 0.314 | 0.527 |
| Microbacteriaceae | 0.39 | 0.52 | 0.66 |  | 0.26 | 0.16 | 0.2 | 0.308 | 0.525 |
| Micrococcaceae | 0.84 | 0.83 | 1 |  | 0.8 | 0.57 | 0.36 | 0.533 | 0.701 |
| Nocardiaceae | 0.02 | 0.01 | 0.03 |  | 0.01 | 0.02 | 0.04 | 0.493 | 0.664 |
| Nocardioidaceae | 0.11 | 0.14 | 0.12 |  | 0.13 | 0.12 | 0.08 | 0.853 | 0.898 |
| Nocardiopsaceae | 0.02 | 0 | 0.01 |  | 0.02 | 0.01 | 0.02 | 0.481 | 0.654 |
| Promicromonosporaceae | 0.04 | 0.01 | 0.03 |  | 0.05 | 0.03 | 0.03 | 0.297 | 0.525 |
| Streptomycetaceae | 0.03 | 0.03 | 0.02 |  | 0.04 | 0.03 | 0.01 | 0.624 | 0.746 |
| Yaniellaceae | 0.67 | 0.95 | 1.25 |  | 0.51 | 0.35 | 0.7 | 0.352 | 0.547 |
| Bifidobacteriaceae | 0.01 | 0.04 | 0.02 |  | 0.02 | 0.07 | 0.01 | 0.403 | 0.59 |
| Coriobacteriaceae | 0.08 | 0.09 | 0.14 |  | 0.07 | 0.06 | 0.08 | 0.440 | 0.625 |
| Solirubrobacterales;f__ | 0 | 0 | 0.03 |  | 0 | 0.01 | 0.03 | 0.035 | 0.431 |
| Patulibacteraceae | 0 | 0 | 0.01 |  | 0.01 | 0 | 0.01 | 0.174 | 0.455 |
| Bacteroidales;f__ | 0.97 | 1.57 | 2.44 |  | 0.66 | 1.01 | 1.23 | 0.159 | 0.455 |
| BS11 | 0.17 | 0.27 | 0.46 |  | 0.1 | 0.16 | 0.22 | 0.091 | 0.431 |
| Bacteroidaceae | 0.71 | 1.22 | 2.26 |  | 0.58 | 0.7 | 1.07 | 0.095 | 0.431 |
| Porphyromonadaceae | 0.15 | 0.25 | 0.48 |  | 0.16 | 0.16 | 0.24 | 0.065 | 0.431 |
| RF16 | 0.23 | 0.24 | 0.44 |  | 0.15 | 0.17 | 0.31 | 0.460 | 0.644 |
| Rikenellaceae | 0.4 | 0.66 | 1.18 |  | 0.38 | 0.43 | 0.53 | 0.080 | 0.431 |
| S24-7 | 0.05 | 0.03 | 0.07 |  | 0.02 | 0.04 | 0.04 | 0.232 | 0.5 |
| [Barnesiellaceae] | 0.01 | 0.01 | 0 |  | 0.01 | 0.02 | 0.01 | 0.660 | 0.77 |
| [Odoribacteraceae] | 0 | 0.01 | 0.01 |  | 0.01 | 0.01 | 0.02 | 0.552 | 0.716 |
| [Paraprevotellaceae] | 0.1 | 0.11 | 0.22 |  | 0.08 | 0.06 | 0.13 | 0.284 | 0.525 |
| p-2534-18B5 | 0.03 | 0.03 | 0.01 |  | 0.03 | 0.02 | 0.01 | 0.150 | 0.455 |
| Cyclobacteriaceae | 0.02 | 0.02 | 0.02 |  | 0.04 | 0.04 | 0.01 | 0.586 | 0.739 |
| Cytophagaceae | 0.02 | 0.03 | 0.11 |  | 0.02 | 0.02 | 0.13 | 0.167 | 0.455 |
| Flammeovirgaceae | 0.01 | 0.02 | 0 |  | 0.02 | 0.03 | 0.01 | 0.409 | 0.59 |
| Cryomorphaceae | 0.01 | 0.02 | 0.01 |  | 0.01 | 0.02 | 0.01 | 0.874 | 0.913 |
| Flavobacteriaceae | 0.15 | 0.19 | 0.13 |  | 0.13 | 0.1 | 0.07 | 0.675 | 0.781 |
| [Weeksellaceae] | 7.29^a^ | 0.17^b^ | 0.42^ab^ |  | 8.66 | 0.07 | 0.38 | 0.040 | 0.431 |
| f__Sphingobacteriaceae | 0.76 | 0.76 | 1.01 |  | 0.74 | 0.26 | 0.27 | 0.328 | 0.527 |
| f__Chitinophagaceae | 12.78 | 23.72 | 14.15 |  | 9.43 | 11.7 | 7.45 | 0.252 | 0.509 |
| JG30-KF-CM45;f__ | 0.07 | 0.04 | 0.05 |  | 0.09 | 0.06 | 0.04 | 0.830 | 0.887 |
| o__YS2;f__ | 0.06 | 0.13 | 0.14 |  | 0.06 | 0.12 | 0.08 | 0.353 | 0.547 |
| Elusimicrobiaceae | 0.01 | 0.01 | 0.01 |  | 0.01 | 0.01 | 0.01 | 0.385 | 0.575 |
| Bacillales;Other | 0.01 | 0 | 0.02 |  | 0.02 | 0.01 | 0.02 | 0.409 | 0.59 |
| Bacillales;f__ | 0.09 | 0.08 | 0.09 |  | 0.06 | 0.1 | 0.09 | 0.788 | 0.869 |
| Bacillaceae | 0.82 | 1.64 | 1.56 |  | 0.59 | 0.31 | 0.56 | 0.079 | 0.431 |
| Paenibacillaceae | 0.04 | 0.01 | 0.04 |  | 0.05 | 0.01 | 0.03 | 0.326 | 0.527 |
| Planococcaceae | 4.27 | 5.94 | 6.89 |  | 2.48 | 1.89 | 1.73 | 0.198 | 0.478 |
| Staphylococcaceae | 1.83 | 2.29 | 2.81 |  | 0.99 | 1.05 | 0.55 | 0.288 | 0.525 |
| [Exiguobacteraceae] | 0.02 | 0.02 | 0.07 |  | 0.01 | 0.01 | 0.12 | 0.885 | 0.918 |
| Lactobacillales;Other | 0.04 | 0 | 0.02 |  | 0.03 | 0.01 | 0.01 | 0.087 | 0.431 |
| Lactobacillales;f__ | 0.07^a^ | 0.00^b^ | 0.00^ab^ |  | 0.05 | 0.01 | 0 | 0.005 | 0.271 |
| Aerococcaceae | 1.45 | 2.08 | 3 |  | 1 | 0.44 | 0.99 | 0.079 | 0.431 |
| Carnobacteriaceae | 2.67^a^ | 0.46b | 0.7^0ab^ |  | 1.26 | 0.23 | 0.08 | 0.005 | 0.271 |
| Enterococcaceae | 0 | 0.01 | 0.01 |  | 0 | 0.01 | 0.01 | 0.175 | 0.455 |
| Lactobacillaceae | 0 | 0.01 | 0.03 |  | 0 | 0.01 | 0.03 | 0.064 | 0.431 |
| Streptococcaceae | 0.94 | 0.46 | 0.39 |  | 1.12 | 0.76 | 0.38 | 0.755 | 0.845 |
| Turicibacteraceae | 0.1 | 0.05 | 0.12 |  | 0.09 | 0.03 | 0.09 | 0.299 | 0.525 |
| Clostridiales;Other | 0.1 | 0.17 | 0.26 |  | 0.08 | 0.12 | 0.05 | 0.146 | 0.455 |
| Clostridiales;f__ | 0.9 | 1.47 | 2.18 |  | 0.63 | 0.88 | 0.64 | 0.095 | 0.431 |
| Christensenellaceae | 0.04 | 0.05 | 0.08 |  | 0.02 | 0.05 | 0.04 | 0.226 | 0.493 |
| Clostridiaceae | 0.21 | 0.23 | 0.47 |  | 0.2 | 0.14 | 0.06 | 0.062 | 0.431 |
| Dehalobacteriaceae | 0 | 0 | 0.03 |  | 0 | 0.01 | 0.02 | 0.049 | 0.431 |
| Lachnospiraceae | 0.57 | 0.74 | 1.18 |  | 0.44 | 0.47 | 0.17 | 0.104 | 0.434 |
| Peptococcaceae | 0.03 | 0.05 | 0.13 |  | 0.02 | 0.02 | 0.11 | 0.083 | 0.431 |
| Peptostreptococcaceae | 0.64 | 0.64 | 0.7 |  | 0.61 | 0.2 | 0.24 | 0.442 | 0.625 |
| Ruminococcaceae | 3.62 | 6.23 | 10.14 |  | 2.46 | 3.64 | 4.51 | 0.095 | 0.431 |
| Veillonellaceae | 0.05 | 0.07 | 0.12 |  | 0.03 | 0.06 | 0.05 | 0.176 | 0.455 |
| [Mogibacteriaceae] | 0.19 | 0.33 | 0.38 |  | 0.13 | 0.15 | 0.16 | 0.203 | 0.478 |
| [Tissierellaceae] | 0.07 | 0.08 | 0.16 |  | 0.06 | 0.05 | 0.11 | 0.217 | 0.488 |
| Erysipelotrichaceae | 0.08 | 0.12 | 0.15 |  | 0.05 | 0.09 | 0.09 | 0.598 | 0.741 |
| c__BD1-5;o__;f__ | 1.3 | 0.06 | 0.05 |  | 2.73 | 0.06 | 0.08 | 0.518 | 0.691 |
| Victivallaceae | 0.17 | 0.33 | 0.48 |  | 0.11 | 0.24 | 0.3 | 0.077 | 0.431 |
| Pirellulaceae | 0.01^a^ | 0.01^a^ | 0.04^b^ |  | 0.01 | 0.01 | 0.02 | 0.017 | 0.394 |
| Rhizobiales;f__ | 0.03 | 0.07 | 0.05 |  | 0.02 | 0.03 | 0.02 | 0.098 | 0.431 |
| Caulobacteraceae | 0.03 | 0.04 | 0.07 |  | 0.02 | 0.03 | 0.02 | 0.086 | 0.431 |
| RF32;f__ | 0.04 | 0.06 | 0.1 |  | 0.06 | 0.05 | 0.08 | 0.356 | 0.547 |
| Beijerinckiaceae | 0 | 0.01 | 0.01 |  | 0 | 0.01 | 0.01 | 0.267 | 0.518 |
| Bradyrhizobiaceae | 0.01 | 0 | 0.05 |  | 0.01 | 0.01 | 0.07 | 0.131 | 0.455 |
| Brucellaceae | 0 | 0.01 | 0 |  | 0.01 | 0.01 | 0.01 | 0.477 | 0.654 |
| Hyphomicrobiaceae | 0.21 | 0.17 | 0.23 |  | 0.21 | 0.12 | 0.14 | 0.611 | 0.744 |
| Methylobacteriaceae | 0.01 | 0.03 | 0.05 |  | 0.01 | 0.02 | 0.05 | 0.254 | 0.509 |
| Phyllobacteriaceae | 0.11 | 0.09 | 0.1 |  | 0.1 | 0.06 | 0.06 | 0.980 | 0.992 |
| Rhizobiaceae | 0.11 | 0.08 | 0.11 |  | 0.13 | 0.08 | 0.02 | 0.302 | 0.525 |
| Rhodobacteraceae | 0.35 | 0.42 | 0.61 |  | 0.32 | 0.22 | 0.31 | 0.327 | 0.527 |
| Acetobacteraceae | 0.00^a^ | 0.00^a^ | 0.01^b^ |  | 0 | 0 | 0.01 | 0.008 | 0.284 |
| Sphingomonadales;f__ | 0.02 | 0.01 | 0.02 |  | 0.02 | 0.02 | 0.02 | 0.611 | 0.744 |
| Erythrobacteraceae | 0.04 | 0.06 | 0.1 |  | 0.02 | 0.04 | 0.06 | 0.106 | 0.434 |
| Sphingomonadaceae | 0.24 | 0.21 | 0.44 |  | 0.17 | 0.13 | 0.34 | 0.326 | 0.527 |
| Burkholderiales;Other | 0.01 | 0.01 | 0.01 |  | 0.01 | 0.01 | 0.02 | 0.992 | 0.992 |
| Alcaligenaceae | 0.79 | 1.81 | 1.6 |  | 0.64 | 0.62 | 0.88 | 0.056 | 0.431 |
| Comamonadaceae | 0.23 | 0.19 | 0.34 |  | 0.2 | 0.12 | 0.12 | 0.142 | 0.455 |
| Oxalobacteraceae | 0.11 | 0.07 | 0.15 |  | 0.05 | 0.09 | 0.14 | 0.165 | 0.455 |
| Neisseriaceae | 0.95 | 0.06 | 0.09 |  | 1.78 | 0.03 | 0.05 | 0.149 | 0.455 |
| Rhodocyclaceae | 0.02 | 0.07 | 0.05 |  | 0.02 | 0.04 | 0.04 | 0.050 | 0.431 |
| Bacteriovoracaceae | 0.03 | 0.02 | 0.04 |  | 0.04 | 0.02 | 0.03 | 0.634 | 0.753 |
| Desulfovibrionaceae | 0.33 | 0.42 | 0.29 |  | 0.49 | 0.48 | 0.12 | 0.348 | 0.547 |
| GMD14H09;f__ | 0.01 | 0.01 | 0 |  | 0.02 | 0.01 | 0 | 0.168 | 0.455 |
| Campylobacteraceae | 0.06 | 0.08 | 0.15 |  | 0.04 | 0.03 | 0.09 | 0.115 | 0.446 |
| Alteromonadales;f__ | 0.01 | 0.04 | 0.03 |  | 0.02 | 0.02 | 0.02 | 0.061 | 0.431 |
| 211ds20 | 0.01 | 0.01 | 0.01 |  | 0.01 | 0.01 | 0.02 | 0.843 | 0.894 |
| Alteromonadaceae | 1.21 | 1.7 | 2.21 |  | 1 | 0.47 | 1.05 | 0.198 | 0.478 |
| HTCC2188 | 0.06 | 0.08 | 0.07 |  | 0.05 | 0.02 | 0.05 | 0.653 | 0.768 |
| Idiomarinaceae | 0.29 | 0.72 | 0.64 |  | 0.3 | 0.16 | 0.33 | 0.120 | 0.449 |
| [Chromatiaceae] | 0.07 | 0.05 | 0.07 |  | 0.08 | 0.04 | 0.05 | 0.820 | 0.883 |
| Cardiobacteriales;f__ | 0.35 | 0.19 | 0.42 |  | 0.41 | 0.21 | 0.77 | 0.569 | 0.724 |
| Cardiobacteriaceae | 0.01 | 0.02 | 0 |  | 0.01 | 0.03 | 0.01 | 0.246 | 0.509 |
| Enterobacteriaceae | 0.03 | 0.03 | 0.1 |  | 0.03 | 0.02 | 0.1 | 0.274 | 0.519 |
| Alcanivoracaceae | 0.01 | 0.01 | 0.01 |  | 0.01 | 0.01 | 0.01 | 0.956 | 0.984 |
| Halomonadaceae | 0.28 | 0.55 | 0.53 |  | 0.14 | 0.26 | 0.57 | 0.198 | 0.478 |
| Oceanospirillaceae | 0.02 | 0 | 0.04 |  | 0.02 | 0.01 | 0.04 | 0.148 | 0.455 |
| Oleiphilaceae | 0.02 | 0.04 | 0.01 |  | 0.02 | 0.04 | 0.01 | 0.307 | 0.525 |
| Pasteurellaceae | 10.74 | 2.3 | 2.7 |  | 11.56 | 2.58 | 2.54 | 0.297 | 0.525 |
| Moraxellaceae | 23.35 | 13 | 10.64 |  | 11.85 | 9.85 | 6.15 | 0.217 | 0.488 |
| Pseudomonadaceae | 2.41 | 3.38 | 4.7 |  | 2.09 | 0.8 | 2.32 | 0.204 | 0.478 |
| Piscirickettsiaceae | 0.09 | 0.18 | 0.2 |  | 0.07 | 0.08 | 0.1 | 0.159 | 0.455 |
| Xanthomonadaceae | 0.31 | 0.46 | 0.6 |  | 0.36 | 0.39 | 0.21 | 0.149 | 0.455 |
| Spirochaetaceae | 0.05^ab^ | 0.04^b^ | 0.13^a^ |  | 0.04 | 0.03 | 0.04 | 0.014 | 0.386 |
| TM7-3;o__;f__ | 0.01 | 0 | 0 |  | 0.01 | 0 | 0.01 | 0.096 | 0.431 |
| Acholeplasmataceae | 0.48 | 1.37 | 1.45 |  | 0.39 | 0.7 | 1.29 | 0.077 | 0.431 |
| Anaeroplasmataceae | 0.03 | 0.05 | 0.12 |  | 0.02 | 0.05 | 0.09 | 0.238 | 0.504 |
| Mycoplasmataceae | 0.73 | 5.99 | 1.38 |  | 0.75 | 6.44 | 1.15 | 0.065 | 0.431 |
| RF39;f__ | 0.05 | 0.08 | 0.12 |  | 0.06 | 0.06 | 0.07 | 0.254 | 0.509 |
| ML615J-28;f__ | 0.07 | 0.15 | 0.22 |  | 0.05 | 0.13 | 0.1 | 0.082 | 0.431 |
| [Cerasicoccaceae] | 0 | 0.01 | 0.01 |  | 0 | 0.01 | 0.01 | 0.137 | 0.455 |
| RFP12 | 0.08 | 0.12 | 0.22 |  | 0.06 | 0.08 | 0.16 | 0.220 | 0.488 |
| Verrucomicrobiaceae | 0.08 | 0.09 | 0.21 |  | 0.08 | 0.06 | 0.12 | 0.122 | 0.449 |
| Deinococcaceae | 1.1 | 0.01 | 0.02 |  | 2.43 | 0.02 | 0.03 | 0.536 | 0.701 |
| Trueperaceae | 0.02 | 0.03 | 0.06 |  | 0.03 | 0.02 | 0.02 | 0.111 | 0.444 |
|  |  |  |  |  |  |  |  |  |  |
| **Genus** |  |  |  |  |  |  |  |  |  |
| Acidimicrobiales;f__;g__ | 0.01 | 0.02 | 0.01 |  | 0.01 | 0.03 | 0.02 | 0.614 | 0.821 |
| Actinomycetales;Other;Other | 0.02 | 0.01 | 0.02 |  | 0.01 | 0.01 | 0.01 | 0.149 | 0.525 |
| Actinomycetales;f__;g__ | 0.01 | 0.02 | 0.01 |  | 0.02 | 0.02 | 0.02 | 0.811 | 0.914 |
| Actinomycetaceae;g__ | 0.06 | 0.05 | 0.07 |  | 0.06 | 0.03 | 0.06 | 0.917 | 0.957 |
| Georgenia | 0.05 | 0.03 | 0.05 |  | 0.07 | 0.04 | 0.03 | 0.718 | 0.878 |
| Brevibacterium | 0.16 | 0.10 | 0.15 |  | 0.14 | 0.15 | 0.08 | 0.270 | 0.581 |
| Cellulomonadaceae;Other | 0.03 | 0.02 | 0.02 |  | 0.02 | 0.04 | 0.02 | 0.657 | 0.843 |
| Cellulomonas | 0.04 | 0.04 | 0.05 |  | 0.05 | 0.02 | 0.04 | 0.705 | 0.866 |
| Demequina | 0.04 | 0.00 | 0.01 |  | 0.06 | 0.01 | 0.02 | 0.468 | 0.754 |
| Corynebacterium | 2.52 | 3.84 | 3.70 |  | 1.84 | 1.09 | 1 | 0.461 | 0.754 |
| Brachybacterium | 0.28 | 0.26 | 0.24 |  | 0.30 | 0.28 | 0.12 | 0.685 | 0.856 |
| Dermacoccaceae;g__ | 0.07 | 0.06 | 0.04 |  | 0.09 | 0.1 | 0.03 | 0.965 | 0.983 |
| Dietzia | 0.30 | 0.32 | 0.25 |  | 0.35 | 0.3 | 0.11 | 0.810 | 0.914 |
| Intrasporangiaceae;Other | 0.04 | 0.04 | 0.03 |  | 0.05 | 0.04 | 0.02 | 0.795 | 0.914 |
| Intrasporangiaceae;g__ | 0.16 | 0.13 | 0.10 |  | 0.16 | 0.05 | 0.06 | 0.704 | 0.866 |
| Phycicoccus | 0.01 | 0.00 | 0.00 |  | 0.01 | 0.01 | 0 | 0.166 | 0.525 |
| Jonesiaceae;g__ | 0.00 | 0.03 | 0.01 |  | 0.01 | 0.04 | 0.01 | 0.205 | 0.526 |
| Kineococcus | 0.01 | 0.00 | 0.01 |  | 0.02 | 0.01 | 0.01 | 0.444 | 0.744 |
| Microbacteriaceae;Other | 0.04 | 0.03 | 0.04 |  | 0.03 | 0.03 | 0.02 | 0.904 | 0.948 |
| Microbacteriaceae;g__ | 0.06 | 0.06 | 0.08 |  | 0.04 | 0.05 | 0.02 | 0.556 | 0.785 |
| Leucobacter | 0.21 | 0.35 | 0.41 |  | 0.17 | 0.11 | 0.19 | 0.242 | 0.567 |
| Pseudoclavibacter | 0.04 | 0.03 | 0.04 |  | 0.03 | 0.03 | 0.03 | 0.801 | 0.914 |
| Rathayibacter | 0.01 | 0.00 | 0.01 |  | 0.01 | 0.01 | 0.01 | 0.168 | 0.525 |
| Salinibacterium | 0.03 | 0.03 | 0.04 |  | 0.02 | 0.03 | 0.02 | 0.763 | 0.903 |
| Micrococcaceae;Other | 0.07 | 0.07 | 0.05 |  | 0.08 | 0.04 | 0.04 | 0.650 | 0.842 |
| Micrococcaceae;g__ | 0.25 | 0.23 | 0.26 |  | 0.22 | 0.16 | 0.11 | 0.959 | 0.983 |
| Arthrobacter | 0.44 | 0.40 | 0.41 |  | 0.49 | 0.33 | 0.22 | 0.835 | 0.923 |
| Kocuria | 0.02 | 0.05 | 0.06 |  | 0.02 | 0.04 | 0.03 | 0.124 | 0.514 |
| Micrococcus | 0.02 | 0.05 | 0.01 |  | 0.02 | 0.04 | 0.01 | 0.086 | 0.475 |
| Nesterenkonia | 0.05 | 0.03 | 0.04 |  | 0.08 | 0.04 | 0.02 | 0.690 | 0.856 |
| Rhodococcus | 0.02 | 0.01 | 0.01 |  | 0.01 | 0.02 | 0.01 | 0.349 | 0.655 |
| Nocardioidaceae;g__ | 0.08 | 0.09 | 0.06 |  | 0.09 | 0.05 | 0.05 | 0.593 | 0.821 |
| Aeromicrobium | 0.03 | 0.05 | 0.03 |  | 0.04 | 0.06 | 0.03 | 0.829 | 0.923 |
| Xylanimicrobium | 0.03 | 0.01 | 0.01 |  | 0.04 | 0.03 | 0.02 | 0.415 | 0.709 |
| Yaniella | 0.67 | 0.95 | 1.07 |  | 0.51 | 0.35 | 0.72 | 0.402 | 0.705 |
| Bifidobacterium | 0.01 | 0.04 | 0.01 |  | 0.02 | 0.07 | 0.01 | 0.543 | 0.785 |
| Coriobacteriaceae;g__ | 0.07 | 0.08 | 0.10 |  | 0.07 | 0.05 | 0.05 | 0.609 | 0.821 |
| Adlercreutzia | 0.01 | 0.00 | 0.01 |  | 0.01 | 0.01 | 0.01 | 0.533 | 0.785 |
| Atopobium | 0.01 | 0.00 | 0.00 |  | 0.01 | 0.01 | 0.01 | 0.814 | 0.914 |
| Solirubrobacterales;f__;g__ | 0.00 | 0.00 | 0.01 |  | 0.00 | 0.01 | 0.01 | 0.192 | 0.525 |
| Patulibacter | 0.00^a^ | 0.00^a^ | 0.01^b^ |  | 0.00 | 0 | 0.01 | 0.008 | 0.475 |
| o__Bacteroidales;f__;g__ | 0.97 | 1.57 | 2.71 |  | 0.66 | 1.01 | 0.93 | 0.070 | 0.475 |
| BS11;g__ | 0.17^a^ | 0.26^ab^ | 0.51^b^ |  | 0.10 | 0.16 | 0.17 | 0.028 | 0.475 |
| Bacteroidaceae;g__ | 0.22 | 0.33 | 0.75 |  | 0.17 | 0.23 | 0.3 | 0.051 | 0.475 |
| 5-7N15 | 0.47^a^ | 0.87^ab^ | 1.67^b^ |  | 0.40 | 0.47 | 0.61 | 0.031 | 0.475 |
| g__Bacteroides | 0.01 | 0.02 | 0.01 |  | 0.02 | 0.01 | 0.02 | 0.637 | 0.838 |
| Porphyromonadaceae;g__ | 0.08 | 0.08 | 0.18 |  | 0.12 | 0.04 | 0.2 | 0.157 | 0.525 |
| Paludibacter | 0.06 | 0.16 | 0.27 |  | 0.04 | 0.16 | 0.13 | 0.054 | 0.475 |
| Parabacteroides | 0.01 | 0.01 | 0.04 |  | 0.01 | 0.02 | 0.02 | 0.268 | 0.581 |
| RF16;g__ | 0.23 | 0.24 | 0.46 |  | 0.15 | 0.17 | 0.28 | 0.354 | 0.655 |
| Rikenellaceae;g__ | 0.40 | 0.66 | 1.22 |  | 0.38 | 0.43 | 0.5 | 0.057 | 0.475 |
| S24-7;g__ | 0.05 | 0.03 | 0.07 |  | 0.02 | 0.04 | 0.04 | 0.232 | 0.56 |
| [Barnesiellaceae];g__ | 0.01 | 0.01 | 0.01 |  | 0.01 | 0.02 | 0.01 | 0.867 | 0.927 |
| Odoribacter | 0.00 | 0.01 | 0.01 |  | 0.01 | 0.01 | 0.02 | 0.552 | 0.785 |
| CF231 | 0.07 | 0.07 | 0.16 |  | 0.05 | 0.05 | 0.09 | 0.158 | 0.525 |
| Paraprevotella | 0.01 | 0.01 | 0.01 |  | 0.03 | 0.01 | 0.01 | 0.865 | 0.927 |
| [Prevotella] | 0.01 | 0.03 | 0.05 |  | 0.02 | 0.02 | 0.04 | 0.215 | 0.533 |
| p-2534-18B5;g__ | 0.03 | 0.03 | 0.01 |  | 0.03 | 0.02 | 0.01 | 0.373 | 0.677 |
| Cyclobacteriaceae;g__ | 0.02 | 0.02 | 0.02 |  | 0.04 | 0.04 | 0.01 | 0.586 | 0.82 |
| Dyadobacter | 0.01 | 0.01 | 0.01 |  | 0.02 | 0.01 | 0.01 | 0.766 | 0.903 |
| Hymenobacter | 0.01 | 0.01 | 0.01 |  | 0.01 | 0.01 | 0.02 | 0.840 | 0.924 |
| Flammeovirgaceae;g__ | 0.01 | 0.02 | 0.00 |  | 0.02 | 0.03 | 0.01 | 0.409 | 0.707 |
| Flavobacteriaceae;g__ | 0.11 | 0.15 | 0.10 |  | 0.12 | 0.08 | 0.07 | 0.427 | 0.719 |
| Capnocytophaga | 0.00 | 0.00 | 0.01 |  | 0.00 | 0.01 | 0.01 | 0.247 | 0.567 |
| Myroides | 0.03 | 0.02 | 0.03 |  | 0.02 | 0.02 | 0.02 | 0.785 | 0.913 |
| [Weeksellaceae];Other | 0.55 | 0.01 | 0.02 |  | 1.16 | 0.01 | 0.03 | 0.272 | 0.581 |
| [Weeksellaceae];g__ | 6.73 | 0.15 | 0.40 |  | 9.04 | 0.07 | 0.34 | 0.145 | 0.525 |
| Sphingobacteriaceae;g__ | 0.57 | 0.61 | 0.85 |  | 0.61 | 0.24 | 0.3 | 0.308 | 0.621 |
| Pedobacter | 0.07 | 0.05 | 0.06 |  | 0.03 | 0.03 | 0.02 | 0.323 | 0.638 |
| Sphingobacterium | 0.12 | 0.11 | 0.14 |  | 0.12 | 0.07 | 0.04 | 0.653 | 0.842 |
| Chitinophagaceae;Other | 0.01 | 0.02 | 0.01 |  | 0.01 | 0.02 | 0.01 | 0.809 | 0.914 |
| Chitinophagaceae;g__ | 12.77 | 23.70 | 19.39 |  | 9.43 | 11.69 | 11.68 | 0.330 | 0.647 |
| JG30-KF-CM45;f__;g__ | 0.07 | 0.04 | 0.04 |  | 0.09 | 0.06 | 0.04 | 0.863 | 0.927 |
| 4C0d-2;o__YS2;f__;g__ | 0.06 | 0.13 | 0.15 |  | 0.06 | 0.12 | 0.08 | 0.353 | 0.655 |
| Streptophyta;f__;g__ | 1.20 | 0.57 | 0.12 |  | 1.77 | 1.03 | 0.1 | 0.613 | 0.821 |
| Elusimicrobiaceae;g__ | 0.01 | 0.01 | 0.01 |  | 0.01 | 0.01 | 0.01 | 0.385 | 0.693 |
| Bacillales;Other;Other | 0.01 | 0.00 | 0.02 |  | 0.02 | 0.01 | 0.01 | 0.161 | 0.525 |
| o__Bacillales;f__;g__ | 0.09 | 0.08 | 0.04 |  | 0.06 | 0.1 | 0.03 | 0.367 | 0.671 |
| Bacillaceae;Other | 0.02 | 0.04 | 0.05 |  | 0.02 | 0.03 | 0.02 | 0.178 | 0.525 |
| Bacillaceae;g__ | 0.43^a^ | 1.10^b^ | 0.82^ab^ |  | 0.33 | 0.22 | 0.46 | 0.047 | 0.475 |
| Anaerobacillus | 0.01 | 0.01 | 0.00 |  | 0.01 | 0.01 | 0 | 0.084 | 0.475 |
| Bacillus | 0.34 | 0.42 | 0.42 |  | 0.24 | 0.18 | 0.17 | 0.542 | 0.785 |
| Marinococcus | 0.01 | 0.05 | 0.05 |  | 0.02 | 0.05 | 0.04 | 0.215 | 0.533 |
| Natronobacillus | 0.00 | 0.01 | 0.01 |  | 0.00 | 0.01 | 0.01 | 0.217 | 0.533 |
| Virgibacillus | 0.00 | 0.01 | 0.01 |  | 0.00 | 0.02 | 0.01 | 0.130 | 0.518 |
| Ammoniphilus | 0.01 | 0.00 | 0.00 |  | 0.01 | 0.01 | 0.01 | 0.540 | 0.785 |
| Paenibacillus | 0.03 | 0.01 | 0.03 |  | 0.04 | 0.01 | 0.02 | 0.236 | 0.565 |
| Planococcaceae;Other | 2.46 | 3.50 | 4.03 |  | 1.37 | 1.08 | 1.26 | 0.188 | 0.525 |
| Planococcaceae;g__ | 0.04 | 0.04 | 0.04 |  | 0.05 | 0.06 | 0.04 | 0.885 | 0.935 |
| Lysinibacillus | 0.20 | 0.15 | 0.17 |  | 0.12 | 0.08 | 0.06 | 0.742 | 0.891 |
| Planomicrobium | 0.58 | 0.47 | 0.51 |  | 0.53 | 0.44 | 0.23 | 0.729 | 0.883 |
| Solibacillus | 0.67 | 0.96 | 1.10 |  | 0.39 | 0.45 | 0.36 | 0.250 | 0.567 |
| Sporosarcina | 0.32^a^ | 0.81^ab^ | 0.75^b^ |  | 0.18 | 0.36 | 0.21 | 0.026 | 0.475 |
| Jeotgalicoccus | 0.95 | 1.32 | 1.26 |  | 0.58 | 0.53 | 0.34 | 0.615 | 0.821 |
| Macrococcus | 0.09 | 0.05 | 0.05 |  | 0.11 | 0.04 | 0.02 | 0.997 | 0.997 |
| Salinicoccus | 0.17 | 0.20 | 0.22 |  | 0.15 | 0.13 | 0.11 | 0.640 | 0.838 |
| Staphylococcus | 0.63 | 0.71 | 0.92 |  | 0.31 | 0.45 | 0.5 | 0.533 | 0.785 |
| Exiguobacterium | 0.02 | 0.02 | 0.03 |  | 0.01 | 0.01 | 0.04 | 0.885 | 0.935 |
| Lactobacillales;Other;Other | 0.04 | 0.00 | 0.01 |  | 0.03 | 0.01 | 0.01 | 0.094 | 0.475 |
| Lactobacillace;g__ | 0.07^a^ | 0.00^b^ | 0.00^b^ |  | 0.05 | 0.01 | 0 | 0.005 | 0.412 |
| LactoAerococcaceae;Other | 0.01^a^ | 0.00^a^ | 0.04^b^ |  | 0.01 | 0.01 | 0.02 | 0.040 | 0.475 |
| LactoAerococcaceae;g__ | 0.88 | 1.52 | 1.76 |  | 0.62 | 0.33 | 0.81 | 0.103 | 0.475 |
| Aerococcus | 0.02 | 0.02 | 0.03 |  | 0.02 | 0.01 | 0.02 | 0.858 | 0.927 |
| Alkalibacterium | 0.02 | 0.02 | 0.01 |  | 0.02 | 0.01 | 0.01 | 0.637 | 0.838 |
| Facklamia | 0.51 | 0.52 | 0.76 |  | 0.36 | 0.14 | 0.42 | 0.690 | 0.856 |
| Carnobacteriaceae;g__ | 0.01 | 0.02 | 0.03 |  | 0.01 | 0.03 | 0.02 | 0.523 | 0.785 |
| Carnobacterium | 0.01 | 0.01 | 0.01 |  | 0.01 | 0.02 | 0.02 | 0.871 | 0.928 |
| Desemzia | 0.03 | 0.05 | 0.05 |  | 0.02 | 0.06 | 0.02 | 0.724 | 0.882 |
| Trichococcus | 2.62^a^ | 0.38^b^ | 0.59^ab^ |  | 1.28 | 0.16 | 0.12 | 0.003 | 0.412 |
| Lactobacillus | 0.00 | 0.01 | 0.01 |  | 0.00 | 0.01 | 0.01 | 0.174 | 0.525 |
| Streptococcus | 0.94 | 0.46 | 0.16 |  | 1.12 | 0.76 | 0.17 | 0.487 | 0.762 |
| Turicibacter | 0.10 | 0.05 | 0.12 |  | 0.09 | 0.03 | 0.09 | 0.299 | 0.61 |
| Clostridiales;Other;Other | 0.10 | 0.17 | 0.27 |  | 0.08 | 0.12 | 0.03 | 0.089 | 0.475 |
| o__Clostridiales;f__;g__ | 0.90 | 1.47 | 2.35 |  | 0.63 | 0.88 | 0.45 | 0.056 | 0.475 |
| Christensenellaceae;g__ | 0.04 | 0.05 | 0.10 |  | 0.02 | 0.05 | 0.03 | 0.083 | 0.475 |
| Clostridiaceae;g__ | 0.11 | 0.12 | 0.25 |  | 0.10 | 0.07 | 0.08 | 0.092 | 0.475 |
| Clostridium | 0.09 | 0.08 | 0.17 |  | 0.08 | 0.06 | 0.05 | 0.141 | 0.525 |
| Proteiniclasticum | 0.01 | 0.02 | 0.01 |  | 0.01 | 0.02 | 0.01 | 0.220 | 0.535 |
| Lachnospiraceae;Other | 0.02 | 0.02 | 0.02 |  | 0.02 | 0.02 | 0.02 | 0.857 | 0.927 |
| Lachnospiraceae;g__ | 0.27 | 0.40 | 0.66 |  | 0.25 | 0.24 | 0.14 | 0.102 | 0.475 |
| Blautia | 0.02 | 0.00 | 0.02 |  | 0.03 | 0.01 | 0.01 | 0.069 | 0.475 |
| Butyrivibrio | 0.07 | 0.06 | 0.09 |  | 0.03 | 0.03 | 0.03 | 0.200 | 0.525 |
| Coprococcus | 0.02 | 0.04 | 0.08 |  | 0.03 | 0.04 | 0.04 | 0.138 | 0.525 |
| Dorea | 0.16 | 0.20 | 0.24 |  | 0.14 | 0.17 | 0.03 | 0.492 | 0.766 |
| Roseburia | 0.01 | 0.01 | 0.04 |  | 0.01 | 0.02 | 0.04 | 0.197 | 0.525 |
| rc4-4 | 0.03^a^ | 0.05^ab^ | 0.15^b^ |  | 0.02 | 0.02 | 0.09 | 0.010 | 0.475 |
| Peptostreptococcaceae;Other | 0.05 | 0.04 | 0.03 |  | 0.07 | 0.03 | 0.02 | 0.831 | 0.923 |
| Peptostreptococcaceae;g__ | 0.58 | 0.60 | 0.63 |  | 0.54 | 0.2 | 0.27 | 0.534 | 0.785 |
| Ruminococcaceae;Other | 0.05 | 0.04 | 0.08 |  | 0.03 | 0.02 | 0.02 | 0.073 | 0.475 |
| Ruminococcaceae;g__ | 3.36 | 5.86 | 9.99 |  | 2.26 | 3.45 | 3.79 | 0.068 | 0.475 |
| Oscillospira | 0.17 | 0.24 | 0.46 |  | 0.15 | 0.13 | 0.15 | 0.049 | 0.475 |
| Ruminococcus | 0.05 | 0.09 | 0.13 |  | 0.03 | 0.05 | 0.01 | 0.071 | 0.475 |
| Phascolarctobacterium | 0.05 | 0.07 | 0.15 |  | 0.03 | 0.06 | 0.03 | 0.055 | 0.475 |
| [Mogibacteriaceae];g__ | 0.10 | 0.23 | 0.27 |  | 0.10 | 0.13 | 0.11 | 0.102 | 0.475 |
| Mogibacterium | 0.09 | 0.10 | 0.11 |  | 0.03 | 0.03 | 0.07 | 0.739 | 0.891 |
| [Tissierellaceae];g__ | 0.03 | 0.03 | 0.10 |  | 0.02 | 0.02 | 0.07 | 0.168 | 0.525 |
| Tissierella_Soehngenia | 0.02 | 0.04 | 0.03 |  | 0.02 | 0.03 | 0.01 | 0.336 | 0.654 |
| _Erysipelotrichaceae;g__ | 0.03 | 0.05 | 0.07 |  | 0.02 | 0.05 | 0.04 | 0.195 | 0.525 |
| Bulleidia | 0.01 | 0.02 | 0.01 |  | 0.01 | 0.02 | 0.02 | 0.391 | 0.694 |
| Erysipelothrix | 0.04 | 0.04 | 0.05 |  | 0.04 | 0.01 | 0.05 | 0.989 | 0.996 |
| RFN20 | 0.00 | 0.00 | 0.01 |  | 0.00 | 0.01 | 0.01 | 0.171 | 0.525 |
| BD1-5;o__;f__;g__ | 1.30 | 0.06 | 0.04 |  | 2.73 | 0.06 | 0.08 | 0.345 | 0.655 |
| Victivallaceae;g__ | 0.17^a^ | 0.33^ab^ | 0.51^b^ |  | 0.11 | 0.24 | 0.27 | 0.038 | 0.475 |
| f__Pirellulaceae;g__ | 0.01^a^ | 0.01^a^ | 0.03^b^ |  | 0.01 | 0.01 | 0.01 | 0.020 | 0.475 |
| Alphaproteobacteria;o__;f__;g__ | 0.03 | 0.07 | 0.05 |  | 0.02 | 0.03 | 0.02 | 0.113 | 0.503 |
| BD7-3;f__;g__ | 0.00 | 0.01 | 0.00 |  | 0.00 | 0.01 | 0.01 | 0.196 | 0.525 |
| Brevundimonas | 0.02 | 0.02 | 0.04 |  | 0.02 | 0.02 | 0.02 | 0.197 | 0.525 |
| Mycoplana | 0.01 | 0.01 | 0.01 |  | 0.01 | 0.01 | 0.02 | 0.992 | 0.996 |
| RF32;f__;g__ | 0.04 | 0.06 | 0.12 |  | 0.06 | 0.05 | 0.06 | 0.158 | 0.525 |
| Beijerinckiaceae;g__ | 0.00 | 0.01 | 0.01 |  | 0.00 | 0.01 | 0.01 | 0.267 | 0.581 |
| Balneimonas | 0.01 | 0.00 | 0.01 |  | 0.01 | 0 | 0.01 | 0.198 | 0.525 |
| Brucellaceae;Other | 0.00 | 0.01 | 0.00 |  | 0.01 | 0.01 | 0.01 | 0.477 | 0.756 |
| Devosia | 0.21 | 0.17 | 0.23 |  | 0.22 | 0.12 | 0.14 | 0.602 | 0.821 |
| Methylobacterium | 0.01 | 0.02 | 0.03 |  | 0.01 | 0.03 | 0.05 | 0.776 | 0.906 |
| Phyllobacteriaceae;Other | 0.01 | 0.02 | 0.01 |  | 0.01 | 0.01 | 0.01 | 0.255 | 0.567 |
| Phyllobacteriaceae;g__ | 0.10 | 0.07 | 0.07 |  | 0.09 | 0.06 | 0.06 | 0.759 | 0.903 |
| Agrobacterium | 0.11 | 0.08 | 0.09 |  | 0.13 | 0.08 | 0.04 | 0.608 | 0.821 |
| Rhodobacteraceae;Other | 0.03 | 0.08 | 0.04 |  | 0.03 | 0.05 | 0.02 | 0.098 | 0.475 |
| Rhodobacteraceae;g__ | 0.22 | 0.22 | 0.37 |  | 0.20 | 0.14 | 0.2 | 0.387 | 0.693 |
| Paracoccus | 0.09 | 0.09 | 0.15 |  | 0.07 | 0.06 | 0.1 | 0.497 | 0.769 |
| Rhodobacter | 0.01 | 0.01 | 0.01 |  | 0.02 | 0.01 | 0.01 | 0.469 | 0.754 |
| o__Sphingomonadales;f__;g__ | 0.02 | 0.01 | 0.02 |  | 0.02 | 0.02 | 0.02 | 0.835 | 0.923 |
| Erythrobacteraceae;Other | 0.03 | 0.04 | 0.05 |  | 0.02 | 0.03 | 0.02 | 0.347 | 0.655 |
| Erythrobacteraceae;g__ | 0.01 | 0.02 | 0.02 |  | 0.01 | 0.02 | 0.01 | 0.316 | 0.63 |
| Sphingomonadaceae;Other | 0.03 | 0.04 | 0.04 |  | 0.05 | 0.04 | 0.02 | 0.621 | 0.825 |
| Sphingomonadaceae;g__ | 0.05 | 0.02 | 0.04 |  | 0.04 | 0.02 | 0.02 | 0.298 | 0.61 |
| Novosphingobium | 0.00 | 0.01 | 0.02 |  | 0.01 | 0.02 | 0.02 | 0.265 | 0.581 |
| Sphingomonas | 0.08 | 0.07 | 0.13 |  | 0.03 | 0.03 | 0.11 | 0.592 | 0.821 |
| Sphingopyxis | 0.07 | 0.05 | 0.04 |  | 0.10 | 0.07 | 0.03 | 0.905 | 0.948 |
| Burkholderiales;Other;Other | 0.01 | 0.01 | 0.01 |  | 0.01 | 0.01 | 0.02 | 0.992 | 0.996 |
| Alcaligenaceae;g__ | 0.30 | 0.61 | 0.56 |  | 0.20 | 0.2 | 0.24 | 0.062 | 0.475 |
| Oligella | 0.46 | 1.15 | 0.98 |  | 0.42 | 0.41 | 0.71 | 0.060 | 0.475 |
| Sutterella | 0.02 | 0.04 | 0.06 |  | 0.02 | 0.04 | 0.05 | 0.516 | 0.785 |
| Comamonadaceae;Other | 0.01 | 0.00 | 0.00 |  | 0.01 | 0.01 | 0 | 0.117 | 0.503 |
| Comamonadaceae;g__ | 0.04 | 0.01 | 0.04 |  | 0.03 | 0.01 | 0.01 | 0.039 | 0.475 |
| Comamonas | 0.17 | 0.16 | 0.26 |  | 0.16 | 0.12 | 0.13 | 0.164 | 0.525 |
| Ramlibacter | 0.00 | 0.01 | 0.01 |  | 0.01 | 0.02 | 0.01 | 0.685 | 0.856 |
| Oxalobacteraceae;Other | 0.03 | 0.02 | 0.03 |  | 0.02 | 0.03 | 0.02 | 0.531 | 0.785 |
| Oxalobacteraceae;g__ | 0.07 | 0.05 | 0.07 |  | 0.04 | 0.06 | 0.04 | 0.249 | 0.567 |
| Janthinobacterium | 0.01 | 0.00 | 0.00 |  | 0.01 | 0 | 0.01 | 0.096 | 0.475 |
| Neisseriaceae;g__ | 0.91 | 0.04 | 0.04 |  | 1.76 | 0.03 | 0.02 | 0.125 | 0.514 |
| Eikenella | 0.02 | 0.00 | 0.00 |  | 0.02 | 0.01 | 0 | 0.080 | 0.475 |
| Kingella | 0.01 | 0.01 | 0.02 |  | 0.01 | 0.01 | 0.02 | 0.462 | 0.754 |
| Rhodocyclaceae;g__ | 0.01^a^ | 0.05^b^ | 0.03^ab^ |  | 0.01 | 0.04 | 0.02 | 0.021 | 0.475 |
| Azoarcus | 0.01 | 0.01 | 0.02 |  | 0.01 | 0.02 | 0.02 | 0.473 | 0.754 |
| Bacteriovoracaceae;g__ | 0.02 | 0.02 | 0.03 |  | 0.03 | 0.02 | 0.02 | 0.754 | 0.901 |
| Desulfovibrionaceae;Other | 0.18 | 0.20 | 0.07 |  | 0.35 | 0.39 | 0.13 | 0.365 | 0.671 |
| Desulfovibrionaceae;g__ | 0.11 | 0.16 | 0.25 |  | 0.10 | 0.11 | 0.13 | 0.182 | 0.525 |
| Lawsonia | 0.04 | 0.06 | 0.01 |  | 0.06 | 0.11 | 0.02 | 0.847 | 0.924 |
| GMD14H09;f__;g__ | 0.01 | 0.01 | 0.00 |  | 0.02 | 0.01 | 0 | 0.168 | 0.525 |
| Myxococcales;f__;g__ | 0.01 | 0.00 | 0.00 |  | 0.01 | 0.01 | 0 | 0.343 | 0.655 |
| Nannocystis | 0.00 | 0.00 | 0.00 |  | 0.00 | 0 | 0.01 | 0.253 | 0.567 |
| Sorangium | 0.00 | 0.00 | 0.00 |  | 0.00 | 0 | 0.01 | 0.253 | 0.567 |
| Spiro;g__ | 0.00 | 0.00 | 0.00 |  | 0.01 | 0.01 | 0 | 0.668 | 0.852 |
| Arcobacter | 0.01 | 0.01 | 0.02 |  | 0.01 | 0.01 | 0.02 | 0.402 | 0.705 |
| Campylobacter | 0.05^a^ | 0.07^ab^ | 0.15^b^ |  | 0.03 | 0.03 | 0.08 | 0.040 | 0.475 |
| Gammaproteobacteria;Other;Other;Other | 0.01 | 0.00 | 0.00 |  | 0.01 | 0 | 0 | 0.116 | 0.503 |
| Gammaproteobacteria;o__;f__;g__ | 0.00 | 0.00 | 0.00 |  | 0.00 | 0.01 | 0 | 0.199 | 0.525 |
| Aeromonadaceae;g__ | 0.00 | 0.00 | 0.00 |  | 0.00 | 0.01 | 0 | 0.472 | 0.754 |
| Succinivibrio | 0.00 | 0.00 | 0.01 |  | 0.00 | 0 | 0.01 | 0.053 | 0.475 |
| o__Alteromonadales;f__;g__ | 0.01 | 0.04 | 0.03 |  | 0.02 | 0.02 | 0.02 | 0.061 | 0.475 |
| 211ds20;g__ | 0.01 | 0.01 | 0.02 |  | 0.01 | 0.01 | 0.02 | 0.548 | 0.785 |
| Alteromonadaceae;Other | 0.00 | 0.00 | 0.01 |  | 0.01 | 0.01 | 0 | 0.054 | 0.475 |
| Alteromonadaceae;g__ | 0.16 | 0.10 | 0.08 |  | 0.20 | 0.13 | 0.04 | 0.793 | 0.914 |
| BD2-13 | 0.86 | 1.33 | 2.15 |  | 0.63 | 0.32 | 0.98 | 0.056 | 0.475 |
| Candidatus Endobugula | 0.04 | 0.08 | 0.10 |  | 0.03 | 0.04 | 0.04 | 0.087 | 0.475 |
| Cellvibrio | 0.13 | 0.11 | 0.09 |  | 0.15 | 0.11 | 0.06 | 0.963 | 0.983 |
| Marinimicrobium | 0.01 | 0.01 | 0.00 |  | 0.01 | 0.01 | 0 | 0.486 | 0.762 |
| Marinobacter | 0.02 | 0.06 | 0.03 |  | 0.02 | 0.03 | 0.03 | 0.060 | 0.475 |
| HTCC | 0.06 | 0.08 | 0.07 |  | 0.05 | 0.02 | 0.05 | 0.646 | 0.841 |
| Idiomarinaceae;g__ | 0.29 | 0.70 | 0.64 |  | 0.30 | 0.16 | 0.32 | 0.124 | 0.514 |
| Pseudidiomarina | 0.00 | 0.02 | 0.00 |  | 0.01 | 0.01 | 0.01 | 0.041 | 0.475 |
| [Chromatiaceae];g__ | 0.07 | 0.05 | 0.07 |  | 0.08 | 0.04 | 0.05 | 0.805 | 0.914 |
| o__Cardiobacteriales;f__;g__ | 0.35 | 0.19 | 0.08 |  | 0.41 | 0.21 | 0.09 | 0.309 | 0.621 |
| Suttonella | 0.01 | 0.01 | 0.00 |  | 0.01 | 0.02 | 0.01 | 0.682 | 0.856 |
| Enterobacteriaceae;Other | 0.01 | 0.01 | 0.00 |  | 0.01 | 0.01 | 0.01 | 0.418 | 0.709 |
| Enterobacteriaceae;g__ | 0.01 | 0.01 | 0.02 |  | 0.01 | 0.01 | 0.02 | 0.460 | 0.754 |
| Erwinia | 0.01 | 0.01 | 0.01 |  | 0.02 | 0.01 | 0.02 | 0.938 | 0.971 |
| Candidatus Portiera | 0.15 | 0.38 | 0.45 |  | 0.11 | 0.19 | 0.56 | 0.116 | 0.503 |
| Haererehalobacter | 0.01 | 0.02 | 0.00 |  | 0.02 | 0.04 | 0 | 0.294 | 0.61 |
| Halomonas | 0.02 | 0.04 | 0.03 |  | 0.02 | 0.02 | 0.02 | 0.198 | 0.525 |
| Kushneria | 0.10 | 0.10 | 0.02 |  | 0.10 | 0.1 | 0.01 | 0.056 | 0.475 |
| Oleibacter | 0.01 | 0.00 | 0.02 |  | 0.02 | 0.01 | 0.01 | 0.283 | 0.597 |
| Oleiphilaceae;g__ | 0.02 | 0.04 | 0.01 |  | 0.02 | 0.04 | 0.01 | 0.208 | 0.526 |
| Aggregatibacter | 10.49 | 1.51 | 2.12 |  | 11.43 | 2.38 | 2.73 | 0.177 | 0.525 |
| Bibersteinia | 0.02 | 0.02 | 0.01 |  | 0.04 | 0.02 | 0.01 | 0.456 | 0.754 |
| Mannheimia | 0.03 | 0.02 | 0.00 |  | 0.02 | 0.03 | 0.01 | 0.346 | 0.655 |
| Pasteurella | 0.19 | 0.74 | 0.54 |  | 0.16 | 1.3 | 0.9 | 0.925 | 0.961 |
| Moraxellaceae;Other | 18.40 | 9.20 | 4.10 |  | 11.08 | 10.47 | 3.45 | 0.188 | 0.525 |
| Moraxellaceae;g__ | 0.28 | 0.16 | 0.20 |  | 0.08 | 0.09 | 0.09 | 0.079 | 0.475 |
| Acinetobacter | 1.45 | 1.95 | 1.66 |  | 0.79 | 0.78 | 0.27 | 0.530 | 0.785 |
| Enhydrobacter | 0.91 | 0.29 | 0.26 |  | 0.76 | 0.35 | 0.46 | 0.254 | 0.567 |
| Moraxella | 1.99 | 1.11 | 1.05 |  | 1.44 | 1.31 | 1.62 | 0.524 | 0.785 |
| Psychrobacter | 0.32 | 0.28 | 0.11 |  | 0.20 | 0.34 | 0.07 | 0.182 | 0.525 |
| Pseudomonadaceae;Other | 0.02 | 0.03 | 0.02 |  | 0.03 | 0.03 | 0.02 | 0.983 | 0.996 |
| Pseudomonadaceae;g__ | 0.47 | 0.59 | 0.97 |  | 0.44 | 0.13 | 0.39 | 0.066 | 0.475 |
| Pseudomonas | 1.91 | 2.76 | 4.16 |  | 1.62 | 0.66 | 1.97 | 0.130 | 0.518 |
| Methylophaga | 0.09 | 0.17 | 0.27 |  | 0.07 | 0.08 | 0.12 | 0.073 | 0.475 |
| Xanthomonadaceae;Other | 0.00 | 0.01 | 0.01 |  | 0.01 | 0.01 | 0.01 | 0.558 | 0.785 |
| Xanthomonadaceae;g__ | 0.01 | 0.01 | 0.01 |  | 0.01 | 0.01 | 0.01 | 0.951 | 0.981 |
| Luteimonas | 0.21 | 0.29 | 0.39 |  | 0.24 | 0.17 | 0.07 | 0.137 | 0.525 |
| Lysobacter | 0.00 | 0.01 | 0.00 |  | 0.01 | 0.01 | 0.01 | 0.552 | 0.785 |
| Stenotrophomonas | 0.09 | 0.15 | 0.19 |  | 0.09 | 0.21 | 0.13 | 0.299 | 0.61 |
| Treponema | 0.05 | 0.04 | 0.10 |  | 0.04 | 0.03 | 0.06 | 0.097 | 0.475 |
| TM7-3;o__;f__;g__ | 0.01 | 0.00 | 0.00 |  | 0.01 | 0 | 0 | 0.030 | 0.475 |
| Acholeplasma | 0.48 | 1.36 | 1.30 |  | 0.39 | 0.7 | 1.38 | 0.082 | 0.475 |
| Anaeroplasmataceae;g__ | 0.01 | 0.01 | 0.03 |  | 0.01 | 0.02 | 0.02 | 0.203 | 0.526 |
| Anaeroplasma | 0.03 | 0.04 | 0.09 |  | 0.01 | 0.04 | 0.07 | 0.156 | 0.525 |
| Mycoplasma | 0.73 | 5.99 | 2.34 |  | 0.75 | 6.44 | 2.99 | 0.080 | 0.475 |
| RF39;f__;g__ | 0.05 | 0.08 | 0.15 |  | 0.06 | 0.06 | 0.06 | 0.091 | 0.475 |
| ML615J-28;f__;g__ | 0.07^a^ | 0.15^b^ | 0.23^b^ |  | 0.05 | 0.13 | 0.1 | 0.046 | 0.475 |
| [Cerasicoccaceae];g__ | 0.00 | 0.01 | 0.01 |  | 0.00 | 0.01 | 0.01 | 0.284 | 0.597 |
| RFP12;g__ | 0.08 | 0.12 | 0.23 |  | 0.06 | 0.08 | 0.15 | 0.193 | 0.525 |
| Akkermansia | 0.08 | 0.08 | 0.20 |  | 0.08 | 0.06 | 0.13 | 0.162 | 0.525 |
| Deinococcus | 1.10 | 0.01 | 0.00 |  | 2.43 | 0.02 | 0.01 | 0.207 | 0.526 |
| B-42 | 0.02 | 0.03 | 0.04 |  | 0.03 | 0.02 | 0.02 | 0.408 | 0.707 |
|  |  |  |  |  |  |  |  |  |  |
| ^1^ Means not sharing a common superscript differ (*P* < 0.05, Dunn’s multiple comparisons test) | | | | | | | | |  |
| ^2^ *P*-values were adjusted for multiple comparisons based on the Benjamini and Hochberg False discovery rate | | | | | | | | |  |
